# Critical contributions of protein cargos to the functions of macrophage-derived extracellular vesicles

**DOI:** 10.1186/s12951-023-02105-9

**Published:** 2023-09-28

**Authors:** Baolong Liu, Phuong Linh Nguyen, Han Yu, Xingzhi Li, Huiren Wang, Jeffrey Price, Meng Niu, Chittibabu Guda, Xiao Cheng, Xinghui Sun, Regis Moreau, Amanda Ramer-Tait, Michael J. Naldrett, Sophie Alvarez, Jiujiu Yu

**Affiliations:** 1 Department of Nutrition and Health Sciences, University of Nebraska-Lincoln, 230 Filley Hall, Lincoln, NE 68583 USA; 2 Department of Food Science and Technology, University of Nebraska-Lincoln, 260 Food Innovation Center, Lincoln, NE 68588 USA; 3 Nebraska Food for Health Center, University of Nebraska-Lincoln, 115 Food Innovation Center, Lincoln, NE 68588 USA; 4 Department of Genetics, Cell Biology and Anatomy, Bioinformatics and Systems Biology Core, University of Nebraska Medical Center, Omaha, NE 68198 USA; 5 Department of Biochemistry, University of Nebraska-Lincoln, N158 Beadle Center, Lincoln, NE 68588-0665 USA; 6 Department of Nutrition and Health Sciences, University of Nebraska-Lincoln, 316E Ruth Leverton Hall, Lincoln, NE 68583 USA; 7 Proteomics and Metabolomics Facility, Nebraska Center for Biotechnology, University of Nebraska-Lincoln, Lincoln, NE 68588 USA

**Keywords:** Extracellular vesicles, Exosomes, M1 macrophages, M2 macrophages, Protein cargos, Tight junction, Colitis

## Abstract

**Background:**

Macrophages are highly plastic innate immune cells that play key roles in host defense, tissue repair, and homeostasis maintenance. In response to divergent stimuli, macrophages rapidly alter their functions and manifest a wide polarization spectrum with two extremes: M1 or classical activation and M2 or alternative activation. Extracellular vesicles (EVs) secreted from differentially activated macrophages have been shown to have diverse functions, which are primarily attributed to their microRNA cargos. The role of protein cargos in these EVs remains largely unexplored. Therefore, in this study, we focused on the protein cargos in macrophage-derived EVs.

**Results:**

Naïve murine bone marrow-derived macrophages were treated with lipopolysaccharide or interlukin-4 to induce M1 or M2 macrophages, respectively. The proteins of EVs and their parental macrophages were subjected to quantitative proteomics analyses, followed by bioinformatic analyses. The enriched proteins of M1-EVs were involved in proinflammatory pathways and those of M2-EVs were associated with immunomodulation and tissue remodeling. The signature proteins of EVs shared a limited subset of the proteins of their respective progenitor macrophages, but they covered many of the typical pathways and functions of their parental cells, suggesting their respective M1-like and M2-like phenotypes and functions. Experimental examination validated that protein cargos in M1- or M2-EVs induced M1 or M2 polarization, respectively. More importantly, proteins in M1-EVs promoted viability, proliferation, and activation of T lymphocytes, whereas proteins in M2-EVs potently protected the tight junction structure and barrier integrity of epithelial cells from disruption. Intravenous administration of M2-EVs in colitis mice led to their accumulation in the colon, alleviation of colonic inflammation, promotion of M2 macrophage polarization, and improvement of gut barrier functions. Protein cargos in M2-EVs played a key role in their protective function in colitis.

**Conclusion:**

This study has yielded a comprehensive unbiased dataset of protein cargos in macrophage-derived EVs, provided a systemic view of their potential functions, and highlighted the important engagement of protein cargos in the pathophysiological functions of these EVs.

**Supplementary Information:**

The online version contains supplementary material available at 10.1186/s12951-023-02105-9.

## Introduction

Macrophages are innate immune cells that play pivotal roles in tissue repair, host defense, and maintenance of homeostasis [1]. They are highly plastic, heterogenous, and capable of rapidly altering their functions in response to environmental stimuli. It is increasingly recognized that stimulus-activated macrophages have a wide spectrum of activated or polarization phenotypes with two extreme opposite states—M1 or classical activation and M2 or alternative activation [2, 3]. Microbial components, such as lipopolysaccharide (LPS), or cytokines, such as interferon-gamma (IFN-γ), induce naïve (M0) macrophages to polarize into M1 macrophages, leading to increased expression of proinflammatory mediators (inducible nitric oxide synthase [iNOS] and cyclooxygenase 2) and release of the cytokines and chemokines (interleukin [IL]-6, IL-12, IL-1β, tumor necrosis factor-alpha [TNF-α], and chemokine C-X-C motif ligand 10 [Cxcl10]) [4]. The resulting proinflammatory M1 macrophages participate in inflammatory responses, pathogen elimination, and antigen presentation. M2 or alternatively activated macrophages are triggered by parasite infection or by cytokines, such as IL-4 or IL-13 [5]. They express high levels of arginase 1 (Arg1), cluster of differentiation (CD)36, CD301b, and mannose receptor C-type 1 (Mrc1, also known as CD206) and are involved in inflammation resolution and tissue remodeling due to their anti-inflammatory phenotypes.

Extracellular vesicles (EVs) are membrane-enclosed particles released by almost every cell type [6, 7]. EVs have been widely appreciated as potent mediators of intercellular communication. They carry proteins, nucleic acids, lipids, and other components from the progenitor cells and travel locally or systemically. When EVs reach their target cells, they either deliver the biomolecules into recipient cells or trigger specific signaling pathways through ligand-receptor interaction, leading to signaling and/or functional changes in the recipient cells. EVs secreted from macrophages have been shown to exert diverse functions under different conditions [6, 8]. They demonstrate beneficial immunoregulatory roles in tissue repair, infectious defense, and other physiological states. However, under pathological conditions, they can promote the progression of a series of diseases such as atherosclerosis, insulin resistance, tissue fibrosis, and cancer.

Currently, the functions of macrophage-derived EVs are primarily attributed to their microRNA cargos [6, 8]. In EVs from other cell types, besides their RNA cargos, their protein and lipid cargos also serve as critical contributors to their physiopathological functions [6]. However, the importance of protein cargos in macrophage-derived EVs remains largely unexplored. Only a few studies [9–11] have indicated that specific protein cargos of macrophage-derived EVs contribute to their functions, implying the potential importance of protein cargos in EVs liberated from differentially activated macrophages. Therefore, in this study, we decided to conduct a comparative proteomics analysis of proteins from M0-, M1-, and M2-EVs to understand the protein signature of EVs from differentially activated macrophages. This approach would reveal the overall landscape of protein cargos of macrophage-derived EVs, as well as their primary functions, allowing us to obtain a systemic view of protein cargos, not just the functions of a few sporadically-selected proteins, in macrophage-derived EVs. We further chose a few typical functions identified from our comparative bioinformatics analyses and subjected them to experimental examinations. Such validation would provide solid evidence to demonstrate the high biological relevance and predictive power of our comparative bioinformatics analyses. The protein landscape of M1- and M2-EVs and their potential functions identified in this study would provide an unprecedented wealth of information and an integral dataset for future research in the area of macrophage-derived EVs.

## Results

### Characterization of macrophage-derived EVs

**Fig. 1:**
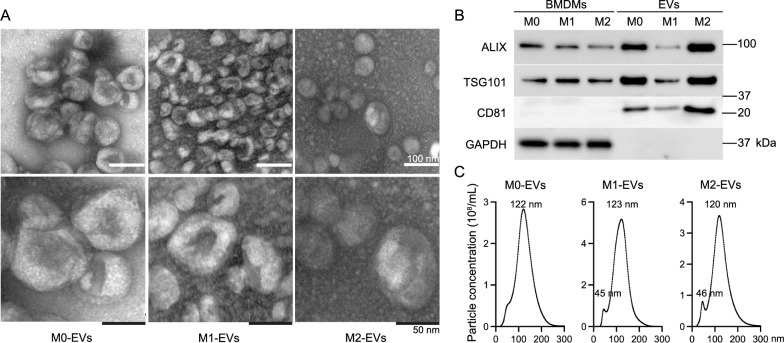
Characterization of macrophage-derived EVs. EVs were isolated from the culture media of M0, M1, and M2 BMDMs and subjected to different analyses. **A** Representative TEM images of M0-, M1-, and M2-EVs. **B** EV validation using immunoblot analysis with antibodies against EV markers. 5 μg proteins from cellular or EV lysates were loaded in each lane. **C** Yield and size distribution of EVs using NanoSight NS300

Naïve M0 mouse bone marrow-derived macrophages (BMDMs) were treated with LPS or IL-4 to induce M1 or M2 macrophages, respectively. Their activating states were confirmed with flow cytometry analysis using antibodies against iNOS and Arg1 (Additional file 1: Fig. S1A), the prototypical markers for M1 and M2 macrophages, respectively [2]. The culture media of M0, M1, and M2 BMDMs were collected to extract EVs (M0-, M1-, and M2-EVs) using the ultracentrifugation protocol [12]. When examined under transmission electron microscopy, all three types of macrophage-derived EVs demonstrated the typical cup-shaped morphology, and their sizes ranged from 50 to 150 nm in diameter (Fig. 1A). Following the guidelines of the International Society for Extracellular Vesicles [7], the macrophage-derived EVs were validated using antibodies against two cytosolic proteins with plasma membrane-binding ability (ALIX and TSG101) and one tetraspanin protein (CD81). All three types of macrophage-derived EVs contained ALIX, TSG101, and CD81, but not glyceraldehyde 3-phosphate dehydrogenase (GAPDH, Fig. 1B), confirming their EV identities at the molecular level. Nanoparticle tracking analysis showed that these three types of macrophage-derived EVs had comparable average diameters of approximately 120 nm (Fig. 1C). Interestingly, the yield of M1-EVs was significantly higher than that of M0-EVs, indicating that EVs were more actively released by M1 macrophages compared to naïve macrophages (Additional file 1: Fig. S1B). The purity of these macrophage-derived EVs was determined by comparing the ratio of EV counts to protein concentration [13]. The ratios of M0-, M1-, and M2-EVs were approximately 0.13–0.3 × 10^10^/μg (Additional file 1: Fig. S1C), comparable to those of EVs from the culture media of cancer cell lines grown in standard cell culture flasks [13].

### Comparative analyses of protein profiles of M0-, M1-, and M2-EVs

Because we focused on the protein cargos of macrophage-derived EVs in this study, we extracted proteins from three biological replicates of M0- and M1-EV pairs, as well as three biological replicates of M0- and M2-EV pairs. The proteins from each EV sample were subjected to quantitative proteomics analysis to obtain their protein profiles (Additional file 2: Table S1, Additional file 3: Table S2). Comparative analysis of protein profiles of M1- and M0-EV pairs revealed that 79 proteins were significantly upregulated and 38 downregulated in M1-EVs, compared to M0-EVs (Additional file 1: Fig. S2A). Principal component analysis (PCA) of the protein profiles of M0- and M1-EV pairs demonstrated a good separation of M0- and M1-EV proteins (Additional file 1: Fig. S2B). Comparison of protein profiles of M2- and M0-EV pairs showed that 53 proteins were significantly enriched and 64 decreased in M2-EVs, compared to M0-EVs (Additional file 1: Fig. S2C). PCA showed that M2-EV proteins were clustered together and separated from M0-EV proteins (Additional file 1: Fig. S2D). The proteins in M1- or M2-EVs that were at least 1.5-fold more abundant than in M0-EVs with p < 0.05 were selected as signature proteins of M1-EVs (73 proteins) or M2-EVs (53 proteins, Additional file 4: Table S3). Volcano plots showed that proteins were significantly changed in M1- or M2-EVs, compared to M0-EVs (Additional file 1: Fig. S2E, F). Interestingly, Cxcl10 and CD40, both highly induced in M1 macrophages [14, 15], were among the most upregulated proteins in M1-EVs (Additional file 1: Fig. S2E). Typical M2 markers Arg1 and macrophage galactose N-acetyl-galactosamine-specific lectin 2 (Mgl2) [5, 16] were prominently enriched in M2-EVs (Additional file 1: Fig. S2F).

**Fig. 2:**
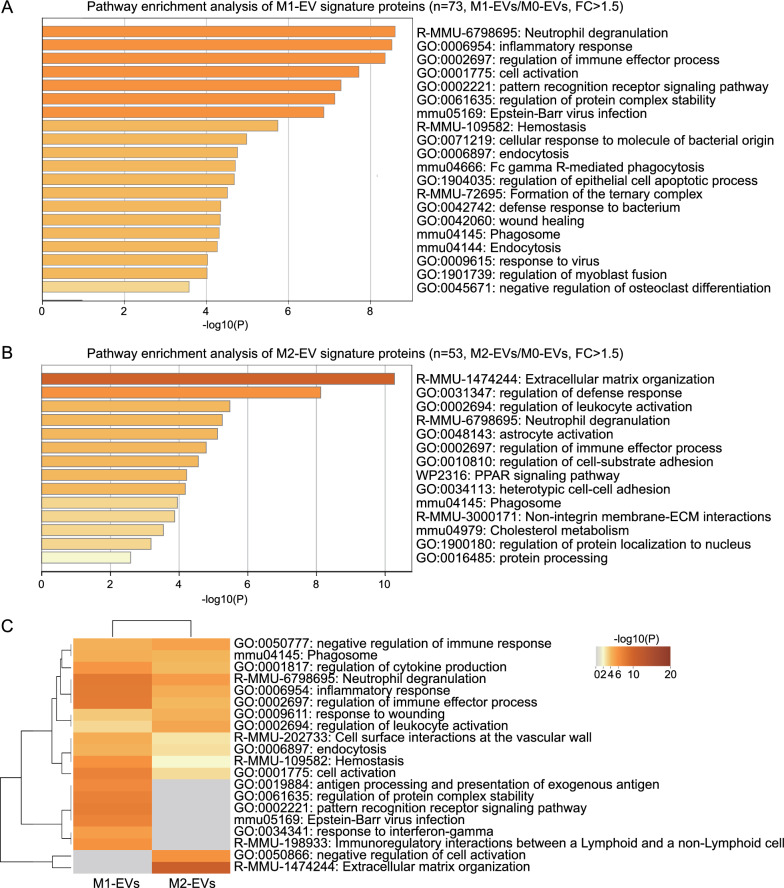
Comparative analyses of protein profiles of M1- and M2-EVs. Total proteins of M0-, M1-, and M2-EVs (3 biological replicates/sample) were subjected to quantitative proteomics analysis. The proteins whose abundances in M1- or M2-EVs were at least 1.5-fold higher than those in M0-EVs with *P* < 0.05 were selected as signature proteins of M1- or M2-EVs. These proteins were used for the pathway enrichment analyses (**A**, **B**) and pathway comparative analysis (**C**). FC: fold change

In order to obtain a systemic view of the protein functions of macrophage-derived EVs, we used the online platform Metascape [17] to identify significantly enriched terms of EV signature proteins in gene ontology (GO) cellular processes, Kyoto Encyclopedia of Genes and Genomes (KEGG) pathways, Reactome gene sets, and WikiPathways. This pathway enrichment analysis showed that the signature proteins of M1-EVs were predominantly involved in proinflammatory terms, such as inflammatory response and pattern recognition receptor (PRR) signaling (Fig. 2A). The proteins significantly enriched in M2-EVs were mainly implicated in immunomodulation and tissue remodeling terms, such as extracellular matrix (ECM) organization and the peroxisome proliferator-activated receptor (PPAR) signaling pathway (Fig. 2B). We conducted further comparative analyses of enriched pathways in M1- and M2-EVs to understand the similarities and divergences between them (Fig. 2C). Some of the signature proteins in M1- and M2-EVs were involved in similar pathways, such as phagocytosis, a well-recognized cellular function of macrophages [18]. However, many M1- and M2-EV signature proteins were involved in separate and distinct pathways. M1-EVs were uniquely enriched in proteins related to antigen processing and presentation, PRR signaling, and response to IFN-γ (Fig. 2C), all of which resembled the primary functions of M1 macrophages [3, 4]. In regard to M2-EVs, they exclusively contained proteins participating in the negative regulation of cell activation and ECM organization (Fig. 2C), simulating the main activities of M2 macrophages [3, 4].

**Fig. 3:**
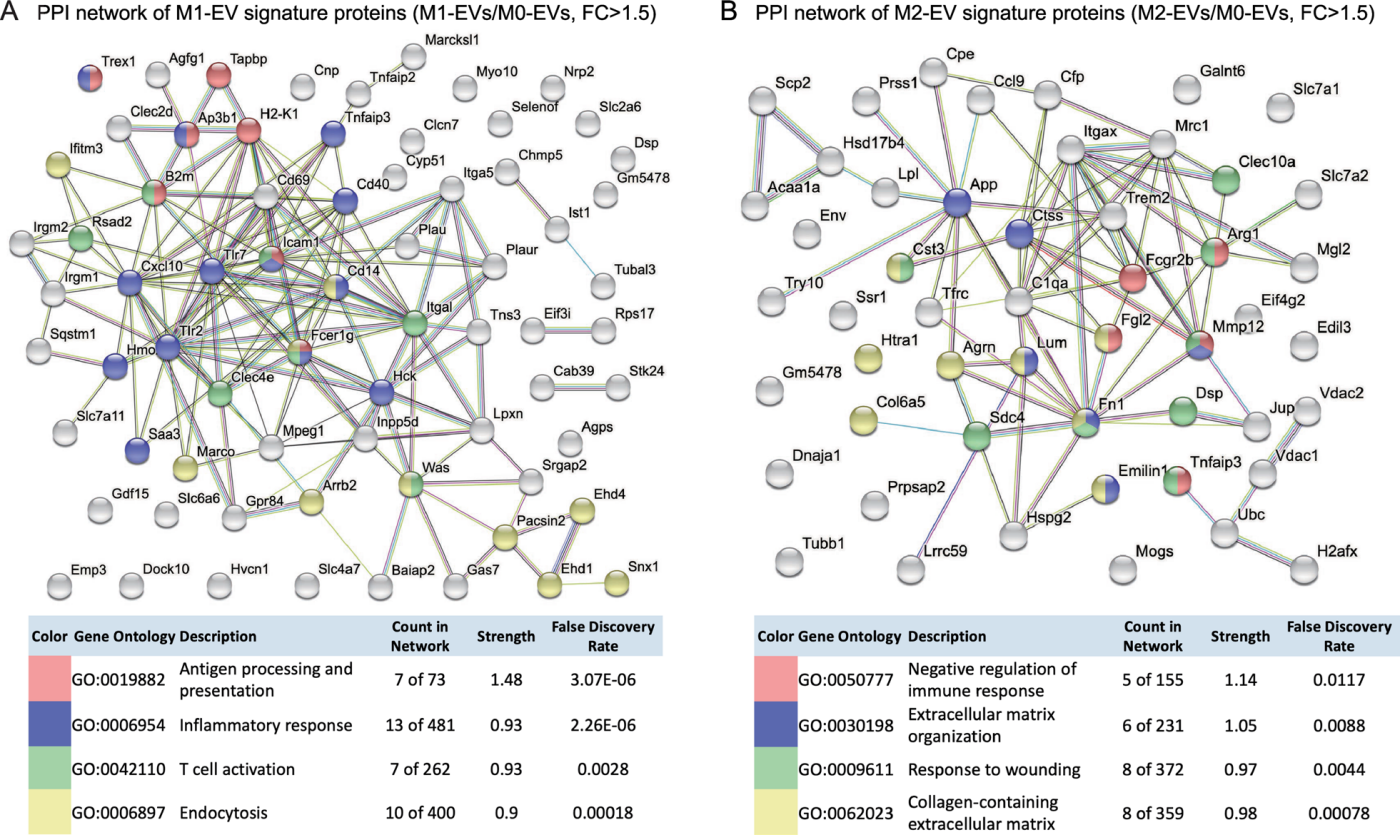
PPI networks of M1-EV and M2-EV signature proteins. PPI analysis was conducted with the signature proteins of M1-EVs (**A**) and M2-EVs (**B**). The major protein clusters within each network were highlighted with different colors. The GO pathways that each protein cluster was enriched were indicated below the PPI networks. The clusters of enriched proteins in M1- or M2-EVs with (a) count in network > 5, (b) strength > 0.9, and (c) false discovery rate < 0.05 in GO terms were highlighted in the network. Count in network: the first number indicates number of proteins in the network that were annotated with a particular term, and the second number indicates number of proteins in total (in the network and in the background) that had this term assigned. Strength: describes the size of the enrichment effect; False discovery rate: describes the significance of the enrichment. FC: fold change

To further understand the functional connections of signature proteins in M1- and M2-EVs, we subjected their signature protein profiles to protein interaction analysis with Search Tool for the Retrieval of Interacting Genes/Proteins (STRING) [19] to reveal protein–protein interaction (PPI) networks. Based on the counts and strength values, the major protein clusters in the M1-EV protein network were found to be highly relevant to pathways involving antigen processing and presentation, inflammatory response, T cell activation, and endocytosis (Fig. 3A), reflecting the pro-inflammatory nature of M1-EV proteins. Meanwhile, the major protein clusters in the M2-EV protein network were intimately associated with pathways involved in negative regulation of immune response, ECM organization, response to wounding, and collagen-containing ECM (Fig. 3B), indicating their involvement in inflammation resolution and tissue remodeling.

Gene set enrichment analysis (GSEA) is a powerful analytical tool originally developed to interpret gene expression profiles [20]. Compared to other analytical approaches that focus only on significantly changed genes, GSEA emphasizes gene sets (groups of genes that share common biological functions) and ranks all the genes in a given data set without setting any arbitrary threshold. GSEA also has been applied in analyzing proteomics data in a cardiomyopathy study [21]. We compared all the identified proteins in M1- or M2-EVs with the existing ontology gene sets (mouse collections) from the Molecular Signature Database (MsigDB) in GSEA. The analysis (using all EV proteins) showed remarkably similar results (Additional file 1: Fig. S3A, B) to those of pathway enrichment and PPI network analyses (using only significantly upregulated proteins). M1-EV proteins were primarily involved in proinflammatory pathways (Additional file 1: Fig. S3A) and M2-EV proteins were mainly related to tissue remodeling (Additional file 1: Fig. S3B). We used the recently developed online tool PSEA-Quant (which uses protein sets from GO and MsigDB to specifically analyze proteomics data [22]) to analyze the total proteins from M1- and M2-EVs. The obtained results (Additional file 1: Fig. S4A, B) were highly consistent with those of our GSEA, pathway enrichment, and PPI network analyses.

Together, the results of pathway enrichment, PPI network, GSEA, and PSEA-Quant analyses were highly consistent and supportive of the notion that protein cargos loaded in macrophage-derived EVs seemed to confer on them functions similar to those of their parental macrophages.

### Comparative analyses of protein signatures of M1- and M2-EVs with their respective parental macrophages

**Fig. 4:**
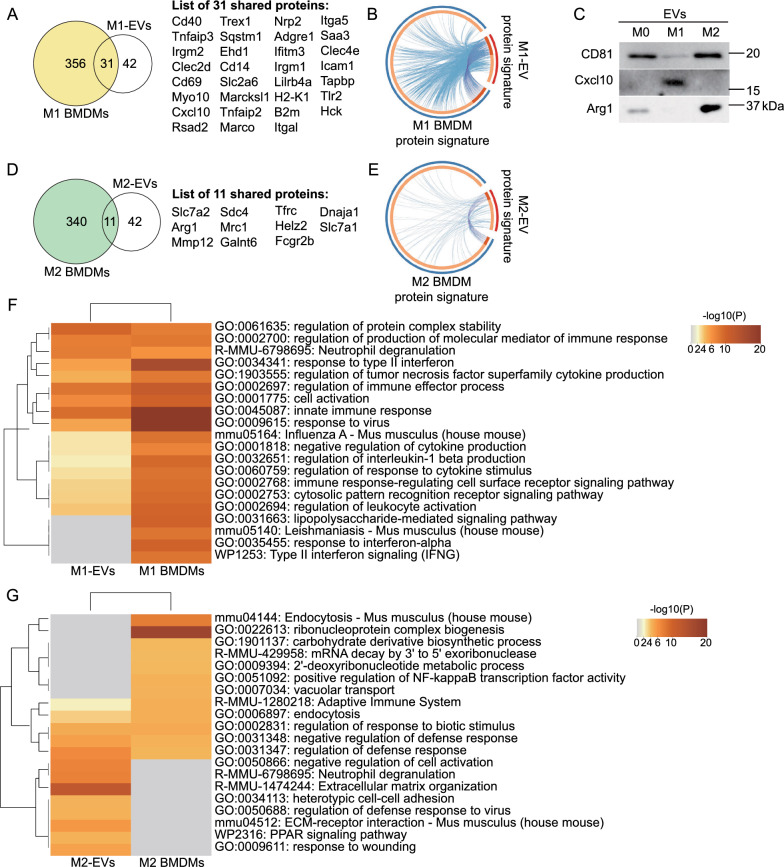
Comparative analyses of protein signatures of M1- and M2-EVs with protein signatures of their respective parental macrophages. **A** Venn diagram showing the overlap of protein signatures of M1-EVs with those of M1 BMDMs. **B** Circos plot depicting the signature proteins and pathways shared between M1-EVs and M1 BMDMs. **C** Immunoblot analysis of Cxcl10 and Arg1. In each lane, 5 μg proteins from EV lysates were loaded. CD81 served as a loading control. **D** Overlap of enriched proteins in M2-EVs with those of M2 BMDMs. **E** Circos plot demonstrating the mutual signature proteins and pathways of M2-EVs and M2 BMDMs. **F** Heatmap showing that M1-EV signature proteins shared 16 of the top 20 pathways of M1 BMDMs. **G** Heatmap indicating that M2-EV signature proteins shared 5 of the top 12 pathways of M2 BMDMs

Next, we sought to understand the extent of the protein signature resemblance between EVs and their parental macrophages. Total proteins of M0, M1, and M2 BMDMs were subjected to quantitative proteomics analysis. Proteins that were at least 1.5-fold more abundant in M1 or M2 BMDMs with p < 0.05 than in M0 BMDMs were considered to be signature proteins of M1 BMDMs (387 proteins) or M2 BMDMs (351 proteins); we used these to conduct the comparative analyses with signature proteins of M1- or M2-EVs, respectively. Thirty-one out of 73 signature proteins in M1-EVs overlapped with proteins that were significantly enriched in M1 BMDMs (Fig. 4A). These include CD40, Cxcl10, and many other proteins involved in inflammatory responses (including serum amyloid A 3 [Saa3], tumor necrosis factor alpha-induced protein 2 [Tnfaip2], and toll-like receptor 2 [Tlr2]). The Circos plot (Fig. 4B) depicted the shared signature proteins and pathways between M1-EVs and M1 BMDMs. CD40 and Cxcl10 are highly induced in M1 macrophages and sometimes considered to be M1 markers [14, 15]. Immunoblot analysis confirmed that Cxcl10 was specifically enriched in M1-EVs, compared to M0- or M2-EVs (Fig. 4C). Thus, Cxcl10 could potentially serve as a new specific protein marker for M1-EVs. Among 53 signature proteins in M2-EVs, 11 were found in the protein signature of M2 BMDMs (Fig. 4D). They include M2 markers (Arg1 and Mrc1/CD206) [5] and proteins related to ECM organization and cell adhesion (syndecan-4 [Sdc4], matrix metallopeptidase 12 [Mmp12], polypeptide N-acetylgalactosaminyltransferase 6 [Galnt6]). The Circos plot (Fig. 4E) showed the signature proteins and pathways that overlapped between M2-EVs and M2 BMDMs. Arg1 was verified to be highly present in M2-EVs, compared to M0- or M1-EVs (Fig. 4C), a finding consistent with the volcano plot analysis of M2-EV signature proteins (Additional file 1: Fig. S2F). Therefore, our data suggested that Arg1 was a new specific protein marker for M2-EVs.

The enriched terms of both parental macrophages and their EVs were combined to run a comparative analysis using the Metascape platform to elucidate the functional similarities and differences between macrophages and their EVs. M1-EV signature proteins shared 16 (or 80%) of the top 20 pathways of the protein signature of M1 BMDMs, most of which were involved in inflammatory responses (Fig. 4F). Of the top 100 enriched terms of M1-EVs and M1 BMDMs, 33% were shared by M1-EVs and their parental cells, 53% were uniquely enriched in M1 BMDMs, and 14% were found exclusively in M1-EVs (Additional file 1: Fig. S5). The overlap of pathways between M2-EV proteins and M2 BMDM proteins was not as striking as the pathway overlap between M1-EV and M1 BMDM proteins. Among the top 20 terms of M2-EVs and M2 BMDMs, M2-EVs shared five (or 42%) of 12 pathways with the protein signature of M2 BMDMs (Fig. 4G). Because there were fewer enriched terms in M2 BMDMs compared to M1 BMDMs, only the top 70 enriched terms from M2-EVs and M2 BMDMs were combined in the comparative analysis. Among them, 10% of the pathways were shared by M2-EVs and their donor cells; 66% belonged only to M2 BMDMs; and 24% were exclusively present in M2-EVs (Additional file 1: Fig. S6). Of note, M2-EV signature proteins, compared to M2 BMDM proteins, were highly enriched in the ECM organization pathway, negative regulation of cell activation, and the PPAR signaling pathway (Fig. 4G and Additional file 1: Fig. S6), suggesting their potential unique roles in these processes.

To further confirm the resemblance between parental macrophages and their EVs, the gene expression profiles of murine M1 and M2 BMDMs obtained in an independent study [23] were used to conduct a comparative analysis with the signature proteins of M1- and M2-EVs. In this new analysis, twenty out of 73 signature proteins in M1-EVs overlapped with genes that were significantly increased in M1 BMDMs (Additional file 1: Fig. S7A); their shared signature proteins and pathways were shown by the Circos plot (Additional file 1: Fig. S7B). Among 53 signature proteins in M2-EVs, 12 were found in the gene signatures of M2 BMDMs (Additional file 1: Fig. S7C); their overlapped signature proteins and pathways were demonstrated in the Circos plot (Additional file 1: Fig. S7D). Pathway analysis showed that M1-EV signature proteins shared 15 of the top 20 pathways (75%) of the M1 BMDM gene signature (Additional file 1: Fig. S7E); M2-EVs shared nine of 18 pathways (50%) of the M2 BMDM gene signature (Additional file 1: Fig. S7F). Therefore, comparing protein profiles of M1-/M2-EVs to gene expression profiles of M1/M2 macrophages led to strikingly similar results to those found in the protein profile comparison between EVs and their parental macrophages. Of note, our macrophage culture conditions were slightly different from those in the gene expression profile study. In that study [23], naïve BMDMs were treated with 100 ng/mL of LPS and 20 ng/mL of INF-γ for 24 h to induce M1 BMDMs, or they were treated with 20 ng/mL of IL4 for 24 h to induce M2 BMDMs. The same condition was used in our study to induce M2 BMDMs; however, to induce M1 BMDMs, we incubated naïve BMDMs with 10 ng/mL LPS for 8 h.

Collectively, comparison of protein profiles of EVs with the protein/gene signatures of their parental macrophages demonstrated that EVs shared only a limited number of proteins with their respective parental macrophages, yet the functions of their proteins manifested high similarity with those of their parental cells. M1-EV signature proteins were predominantly enriched in the inflammatory process, reflecting an M1-like profile, and M2-EV signature proteins shared many features of M2 macrophages and thus showed an M2-like phenotype, with further enrichments of proteins related to specific pathways.

### Protein cargos of M1-EVs induced M1 polarization and activated T lymphocytes

**Fig. 5:**
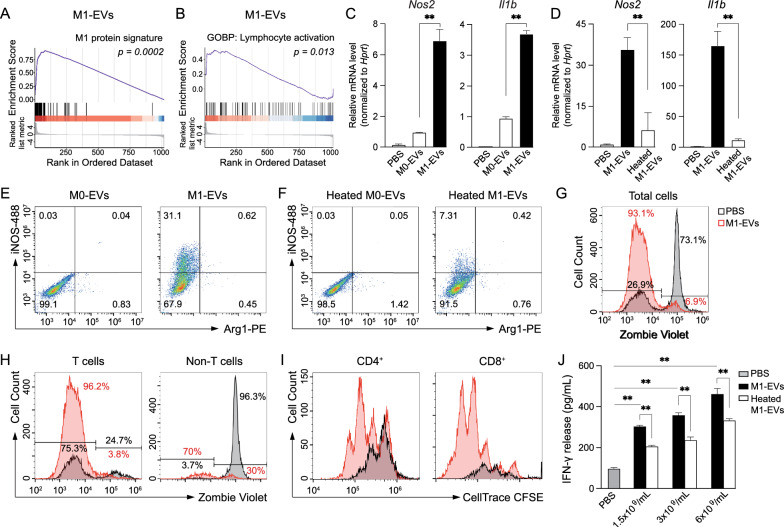
Protein cargos in M1-EVs induced M1 polarization and stimulated T lymphocytes. **A** GSEA showed that M1-EVs were significantly enriched with proteins related to M1 protein signature. **B** GSEA suggested that M1-EV proteins positively correlated with lymphocyte activation. **C** Expression of M1 marker genes in naïve macrophages treated with 1.5 × 10^9^/mL of M0- or M1-EVs for 8 h. **D** Expression of M1 marker genes in naïve macrophages treated with 1.5 × 10^9^/mL of regular or heated M1-EVs for 8 h. EVs were heated at 95 °C for 10 min to denature the proteins. **E**, **F** Flow cytometry analysis showing the protein levels of iNOS and Arg1 in BMDMs treated with 6 × 10^9^/mL of regular (**E**) or heated (**F**) M0- and M1-EVs for 8 h. **G**–**J** Murine splenocytes were primed with anti-CD3 antibody (1 µg/mL) in the absence or presence of 1 × 10^10^/mL M1-EVs for 72 h, followed by flow cytometry analysis and cytokine measurement. **G**–**H** Zombie Violet staining to assess viability of total splenocytes (**G**), T cells (CD4^+^ and/or CD8^+^) and non-T cells (CD4 and CD8 negative, **H**). **I** Proliferation of CD4^+^ and CD8^+^ lymphocytes measured using CellTrace CFSE dye. **J** IFN-γ release from anti-CD3-primed splenocytes treated with regular or heated M1-EVs. Data presented as mean ± STD (N = 3). **P < 0.01

Because GSEA allows us to create new reference sets, we used the signature proteins of M1 BMDMs to create a protein set called “M1 protein signature,” and we used the significantly upregulated genes in M1 BMDMs [23] to create a gene set “M1 gene signature” in GSEA. When total proteins of M1-EVs were compared with these new reference sets, they were found to be highly related to the M1 protein signature (Fig. 5A) or M1 gene signature (Additional file 1: Fig. S7G). In addition, the results from pathway enrichment, PPI network, GSEA, and PSEA-Quant analyses (Figs. 2, 3, Additional file 1: Fig. S3, S4) all indicated that M1-EV proteins were highly related to lymphocyte activation. The enrichment of the “lymphocyte activation” gene set in the ranked M1-EV proteins in GSEA was further visualized in Fig. 5B.

It has been reported that M1-EVs from LPS-treated RAW264.7 cells were capable of repolarizing IL-4-induced M2 BMDMs into M1 macrophages [24] and that M1 BMDM-derived EVs promoted expression of IFN-γ in T lymphocytes [25]. The functions of M1-EVs identified in these previous studies supported the results of our bioinformatic analyses, although the responsible active cargos in M1-EVs were not determined. Therefore, we first sought to validate the role of M1-EVs in macrophage polarization. M1- or M0-EVs were incubated with naïve BMDMs, followed by gene expression analysis. M1-EVs dramatically enhanced expression in BMDMs of M1 marker genes, including the *Nos2* (encoding iNOS protein) and *Il1b* genes [3], compared to phosphate-buffered saline (PBS) or M0-EV-treated cells (Fig. 5C). The transcription of the M2 marker *Arg1* gene was not affected by M1-EVs (Additional file 1: Fig. S8A), indicating the specificity of M1-EVs in inducing M1 macrophages. Flow cytometry analysis further confirmed that M1-EVs induced the protein level of iNOS, but not that of Arg1 (Fig. 5E). Our results were consistent with conclusions of the previous study [24] and validated the results of our bioinformatic analyses. Considering that our bioinformatic analyses were based on M1-EV proteins, we assessed the role of M1-EV proteins in inducing M1 polarization. M1-EVs were heated at 95 °C for 10 min to denature the proteins. Even though most proteins in the heated M1-EVs lost function, their uptake by naïve BMDMs was comparable to the uptake of untreated M1-EVs (Additional file 1: Fig. S8B). However, heated M1-EVs largely failed to induce iNOS protein levels (Fig. 5F) or to enhance the mRNA levels of the *Nos2* and *Il1b* genes (Fig. 5D). Therefore, the protein cargos in M1-EVs played a key role in inducing M1 polarization.

Next, we assessed the effects of M1-EVs on lymphocyte activation. Incubation of M1-EVs with splenocytes from C57BL/6J mice led to increased release of proinflammatory cytokines IFN-γ and TNF-α in a dose-dependent manner (Additional file 1: Fig. S9A, B), suggesting that M1-EVs activated splenocytes. Heat treatment of M1-EVs abolished their stimulatory effects on splenocytes (Additional file 1: Fig. S9A, B), which pointed to the critical role of M1-EV proteins in such functions. To further investigate the effects of M1-EVs specifically on T cells, splenocytes were stimulated with soluble anti-CD3 antibody [26] and concomitantly treated with M1-EVs. Staining of a fixable viability marker Zombie Violet dye indicated that M1-EVs dramatically promoted splenocyte viability in culture (Fig. 5G), as well as the viability of total T cells (Fig. 5H). Proliferation of T lymphocytes was monitored using 5-(and-6)-carboxyfluorescein diacetate succinimidyl ester (CFSE) labeling. Increased cell counts and number of divisions were observed for both CD4^+^ and CD8^+^ T cells after M1-EV treatment compared to control cells (Fig. 5I), illustrating promotion of CD3-primed T cell proliferation by M1-EVs. Lastly, M1-EVs dose-dependently enhanced the release of IFN-γ from anti-CD3-stimulated splenocytes and this effect was significantly curbed by heat treatment of M1-EVs (Fig. 5J). Collectively, our data not only confirmed results of the previous study [25] showing that M1-EVs promoted IFN-γ production in T cells, but also revealed that M1-EVs fostered the viability and proliferation of T lymphocytes. All these functions of M1-EVs were primarily mediated through their protein cargos.

### Protein cargos of M2-EVs induced M2 polarization in naïve BMDMs and protected tight junction from disruption in Caco-2 cells

**Fig. 6:**
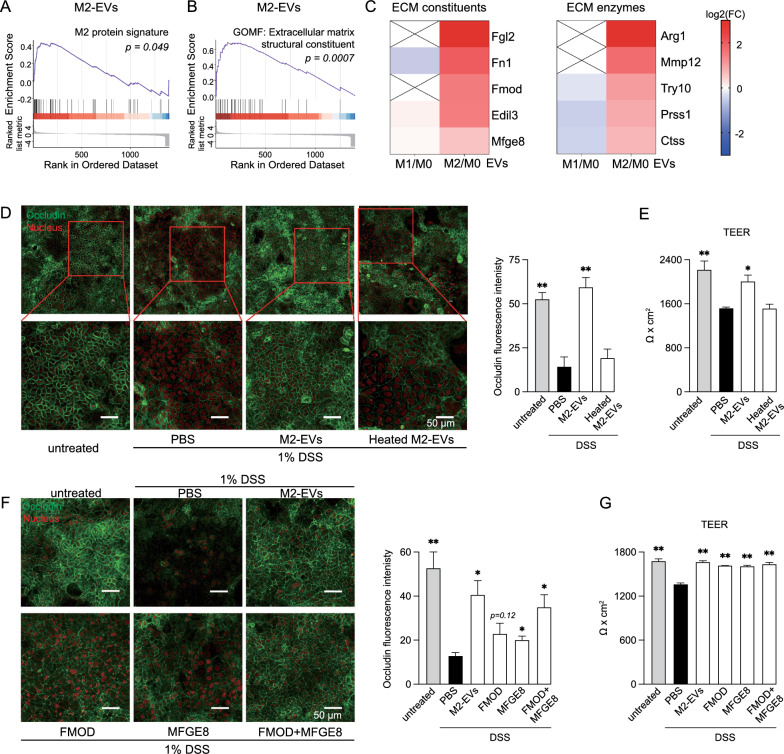
Proteins in M2-EVs were the key components in protecting tight junction structure and barrier integrity from disruption in Caco-2 cells. **A** GSEA showed that M2-EV proteins were highly related to M2 protein signature. **B** GSEA suggested high enrichment of M2-EV proteins in ECM structural constituent. **C** Heatmap showing the relative abundance of representative ECM constituents and enzymes in signature proteins of M2-EVs, compared to those of M1-EVs. **D** Representative images of occludin IF of Caco-2 cells and quantification of occludin signal intensity. The differentiated Caco-2 monolayers were treated with PBS or regular or heated M2-EVs in PBS (6 × 10^9^/mL) for 48 h in the presence of 1% DSS. **E** M2-EVs prevented DSS-induced TEER reduction, but heated M2-EVs lost such protective effects. The differentiated Caco-2 monolayers were pretreated with PBS or regular or heated M2-EVs in PBS (6 × 10^9^/mL) for 36 h, followed by treatment with 1.5% DSS for 48 h. **F** Representative images of occludin IF of Caco-2 cells and quantification of occludin signal intensity. The differentiated Caco-2 cells were treated with PBS, M2-EVs in PBS (6 × 10^9^/mL), FMOD (3 µg/mL), MFGE8 (3 µg/mL), or FMOD (1.5 µg/mL) and MFGE8 (1.5 µg/mL) together for 48 h in the presence of 1% DSS. **G** FMOD and MFGE8 proteins protected cells from DSS-induced TEER reduction. The differentiated Caco-2 monolayers were pretreated with PBS, M2-EVs in PBS (6 × 10^9^/mL), FMOD (3 µg/mL), MFGE8 (3 µg/mL), or FMOD (1.5 µg/mL) and MFGE8 (1.5 µg/mL) together for 36 h, followed by treatment with 1.5% DSS for 48 h. *P < 0.05 and **P < 0.01 relative to cells treated with DSS alone (black bar)

A similar approach was used in GSEA to assess the potential correlation of M2-EV proteins with their parental cell proteins. The signature proteins and genes [23] of M2 BMDMs were used to create “M2 protein signature” and “M2 gene signature” sets, respectively. M2-EV proteins were found to be highly related to the M2 protein signature (Fig. 6A) and the M2 gene signature (Additional file 1: Fig. S7H). M2 BMDM-derived EVs have been shown to induce M2 polarization in M1 BMDMs possibly through synergistic effects of their cytokine cargos [9], supporting the results of our bioinformatic analyses. For validation purposes, naïve BMDMs were incubated with M0- or M2-EVs, followed by gene expression analysis. The *Arg1*, *Cd36*, and *Ccl17* genes are highly induced in M2 macrophages and often considered to be M2 markers [3, 27]. M2-EVs were capable of increasing mRNA levels of these genes (Additional file 1: Fig. S10A) but failed to induce expression of the *Nos2* gene (Additional file 1: Fig. S10B), suggesting that M2-EVs specifically induced M2 polarization in naïve BMDMs. Flow cytometry analysis confirmed that M2-EVs enhanced the protein level of M2 marker Arg1 but not that of M1 marker iNOS (Additional file 1: Fig. S10C). Although the uptake of heated M2-EVs by BMDMs was comparable to that of untreated M2-EVs (Additional file 1: Fig. S10D), heat-treated M2-EVs failed to induce either the protein level of Arg1 (Additional file 1: Fig. S10E) or expression of the *Arg1* and *Cd36* genes (Additional file 1: Fig. S10F). Together, our cell culture tests confirmed that M2-EVs specifically induced M2 polarization in naïve BMDMs through their protein cargos.

Our pathway enrichment, PPI network, GSEA, and PSEA-Quant analyses (Fig. 2, 3, Additional file 1: Fig. S3, 4) all pointed to the important involvement of M2-EV proteins in ECM regulation. In GSEA, the ECM structural constituent was the top ontology term identified with M2-EV proteins (Fig. 6B). These bioinformatic findings led us to experimentally examine the role of M2-EVs in ECM regulation. ECM, primarily comprised of fibrous proteins (such as collagens and elastin) and glycoproteins (such as fibronectin and laminin), not only provides structural scaffolds for cells but also participates in regulation of tissue function and homeostasis [28]. The comparative proteomics analysis showed that signature proteins of M2-EVs were enriched with a variety of ECM constituents and enzymes, compared with those of M1-EVs (Fig. 6C). Some of the ECM enzymes such as Mmp12 and trypsin 1 (Prss1) have been reported to involve collagen degradation [29, 30]. Thus, we first examined whether ECM enzymes in M2-EVs were involved in ECM degradation. M2-EVs were sonicated or lysed to release their protein cargos, followed by incubation with a collagen mixture. Although the positive control collagenase effectively degraded collagens, proteins in M2-EVs had a marginal effect on collagen degradation (Additional file 1: Fig. S11A, B).

ECM regulates cell–cell junction positioning and promotes tight junction formation [31, 32]. Considering the enrichment of ECM constituents in M2-EVs, we hypothesized that M2-EVs possibly engaged in barrier functions. Colorectal adenocarcinoma Caco-2 cells were selected as the cellular model because they spontaneously differentiate into a polarized epithelial monolayer with tight junction structure when reaching confluence [33]. Because dextran sulfate sodium (DSS) was shown to disrupt the tight junction structure of the Caco-2 monolayer [34], we treated the differentiated Caco-2 monolayer with DSS with or without M2-EVs. Consistent with results of the previous study [34], DSS disrupted the tight junction mesh structure of Caco-2 cells, as demonstrated by immunofluorescence (IF) staining using an antibody against the tight junction protein occludin (Fig. 6D). Remarkably, M2-EVs largely protected the tight junction structure from disruption by DSS, which was abolished by heat treatment of M2-EVs (Fig. 6D). IF staining using an antibody against claudin1, another key protein component of tight junction [35], showed a similar pattern (Additional file 1: Fig. S12A), further confirming that M2-EVs, through their protein cargos, were critically engaged in maintenance of tight junction structure.

Transepithelial electrical resistance (TEER) is a measurement of electrical resistance across a cellular monolayer. Because of its high sensitivity and non-invasiveness, it is a widely used quantitative method to evaluate the integrity of tight junction in cultured epithelial monolayers [36]. Consistent with the IF staining results of tight junction proteins, DSS treatment significantly reduced TEER of Caco-2 monolayers, compared to untreated cells (Fig. 6E). Remarkably, M2-EVs, but not heated M2-EVs, blocked such reduction (Fig. 6E), further supporting the notion that proteins in M2-EVs were the key components contributing to the maintenance of barrier integrity.

In M2-EVs, fibromodulin (FMOD), a small leucine-rich glycoprotein involved in ECM composition, has been shown to modulate expression of tight junction proteins and its depletion aggravated DSS-induced destruction of the epithelial barrier in mice [37]. Milk fat globule epidermal growth factor (EGF) factor 8 protein (MFGE8), a cysteine-rich secretory glycoprotein, maintained the integrity of the epididymal epithelium [38]. Administration of recombinant MFGE8 preserved colon integrity in DSS-induced colitis mice [39]. The presence of both FMOD and MFGE8 in M2-EVs was verified by immunoblot using their specific antibodies (Additional file 1: Fig. S12B). Remarkably, recombinant FMOD and MFGE8 proteins partially rescued the tight junction structure of Caco-2 cells from disruption by DSS, as shown by IF staining of occludin (Fig. 6F) and claudin1 (Additional file 1: Fig. S12C). Cotreatment of Caco-2 cells with FMOD and MFGE8 proteins synergistically reserved their tight junction structure in the presence of DSS (Fig. 6F, Additional file 1: Fig. S12C). Interestingly, either FMOD or MFGE8 alone was able to largely curb the decreased TEER caused by DSS treatment, and these two proteins together reversed DSS-triggered TEER decrease (Fig. 6G). Together, it seemed that ECM components like FMOD and MFGE8 in M2-EVs worked together to maintain the tight junction structure and barrier integrity of epithelial cells.

### M2-EVs protected mice from DSS-induced colitis

Inflammatory bowel disease (IBD), consisting of Crohn’s disease and ulcerative colitis, is characterized by chronic and recurrent inflammation of the digestive tract [40]. Compromised integrity of the epithelial barrier has often been observed in IBD patients [41]. Because our cell culture data suggested that proteins in M2-EVs induced polarization of anti-inflammatory M2 macrophages in naïve macrophages and protected Caco-2 cells from disruption of the tight junction structure, we hypothesized that M2-EV proteins may have beneficial functions in the management of IBD.

**Fig. 7:**
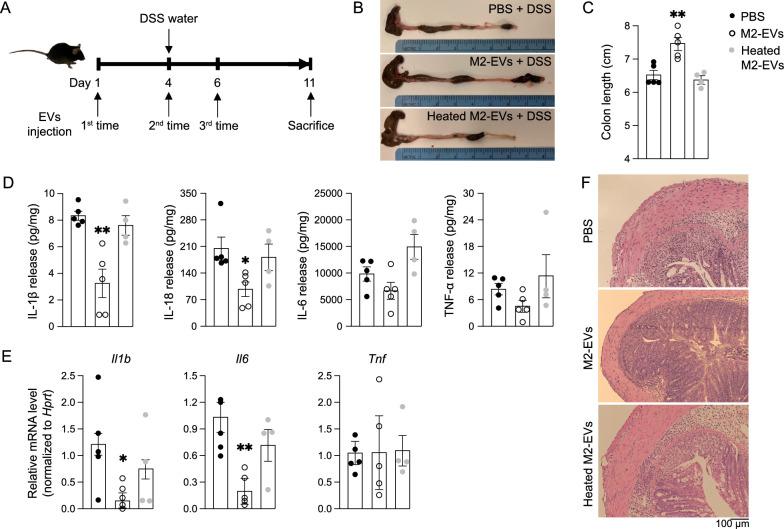
M2-EVs protected mice from DSS-induced colitis through their protein cargos. **A** Schematic diagram showing the experimental procedure. 2-month-old male C57BL/6J mice were intravenously injected with PBS or regular or heated M2-EVs in PBS (3 × 10^8^/g) on day 1, 4, and 6. All mice were given 1.5% (w/v) DSS in drinking water from day 4–11 and sacrificed on day 11. N = 4–5/group. **B** Representative colon images of colitis mice that received PBS or regular or heated M2-EVs in PBS. **C** M2-EVs, but not heated M2-EVs, prevented the shortened colon length in mice induced by DSS treatment. **D** The levels of cytokines in the media of ex vivo cultured colonic tissues. Released cytokine was normalized to the protein concentration of tissue lysates. **E** Expression of pro-inflammatory cytokine genes in colon tissues. **F** Representative images of H&E-stained sections of colon tissues. In the bar graphs, each dot represents one mouse. Data presented as mean ± SEM. * *P* < 0.05 and ** *P* < 0.01 relative to the control colitis mice received PBS (bar with black dots)

Acute colitis was induced in C57BL/6J mice by administering 1.5% (w/v) DSS in drinking water for 7 days; PBS, M2-EVs in PBS, or heated M2-EVs in PBS were intravenously injected into the mice at the indicated time points (Fig. 7A). M2-EVs, but not heated M2-EVs, significantly prevented the shortened colon length in mice induced by DSS treatment (Fig. 7B, C). Measurement of inflammatory cytokine levels in the media of ex vivo cultured colon tissues showed that M2-EV administration significantly reduced release of cytokines IL-1β and IL-18, but heat treatment of M2-EVs abolished such effects (Fig. 7D). At the transcription level, M2-EVs inhibited expression of the *Il1b* and *Il6* genes, whereas heated M2-EVs had marginal effects (Fig. 7E). At the pathological level, hematoxylin and eosin (H&E)-stained colon sections demonstrated that M2-EVs, but not heated M2-EVs, inhibited immune cell infiltration and reserved the intactness of the colon’s mucosa layer, compared to DSS-treated mice that received PBS only (Fig. 7F). Independent animal studies using more mice further confirmed that M2-EV administration strongly protected the colon from colitis development (Additional file 1: Fig. S13A–C). Therefore, it seems that M2-EVs had potent protective functions in the colon with colitis, primarily mediated through their protein cargos.

Considering these remarkable protective properties of M2-EVs in colitis, we assessed whether intravenously injected M2-EVs could physically accumulate in the colon. The membrane proteins of M2-EVs were covalently labeled with a fluorescent dye in near infrared ranges from an ExoGlow-Vivo EV labeling kit. The near infrared fluorescence spectrum of this dye enables deep tissue illumination and eliminates background from auto-fluorescence. The labeled M2-EVs were intravenously given to C57BL/6J mice with DSS-induced colitis, their significant accumulation were found in the liver, spleen, and lung (Additional file 1: Fig. S14A, B), consistent with many studies reporting that EVs often accumulate in these tissues after intravenous injection [42–44]. Interestingly, M2-EVs also markedly accumulated in the colon, as well as in the cecum and upper gastrointestinal tract (Additional file 1: Fig. S14A, B). Such colonic localization likely helped M2-EVs exert their protective functions in the colon.

**Fig. 8:**
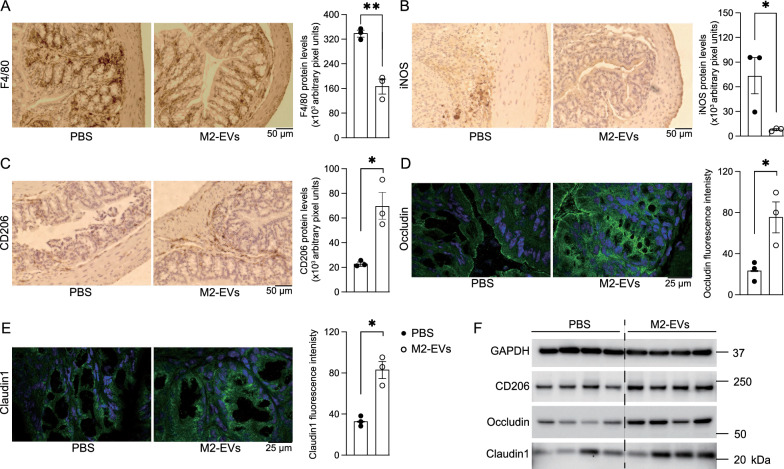
M2-EVs promoted polarization of M2 macrophages and improved barrier functions in DSS-induced colitis. The mice from Additional file 1: Fig. S13 were subjected to further analysis. N = 3–4/group. **A**–**C** Antibodies against macrophage marker F4/80 (**A**), M1 marker iNOS (**B**), and M2 marker CD206 (**C**) were used to conduct IHC of colon sections. Their representative images and quantifications were shown. **D**, **E** Antibodies against tight junction protein occludin (**D**) and claudin1 (**E**) were used to conduct IHF of colon tissues. Their representative images and quantifications were shown. **F** Immunoblot analysis of colon tissues. GAPDH served as a loading control. In the bar graphs, each dot represents one mouse. Data presented as mean ± SEM. *P < 0.05 and **P < 0.01 relative to the control colitis mice received PBS (bar with black dots)

Finally, we investigated the effects of M2-EVs on macrophage polarization and tight junction structure in colitis. Immunohistochemistry (IHC) staining using an antibody against F4/80 (macrophage marker [45]) showed that M2-EV treatment suppressed infiltration of macrophages (Fig. 8A). Regarding the macrophage polarization status, M2-EVs reduced the level of the M1 marker, iNOS protein (Fig. 8B), while enhancing the level of the M2 marker, Mrc1/CD206 protein (Fig. 8C), suggesting that M2-EV administration drastically promoted polarization of intestinal macrophages into the M2 state. Immunohistofluorescence (IHF) of colon sections using anti-occludin antibody demonstrated that M2-EVs not only increased the levels of tight junction proteins, but also reserved the tight junction structure (Fig. 8D). Claudin1 IHF of colon sections showed similar results (Fig. 8E). Consistently, immunoblot analysis showed that levels of both occludin and claudin1, as well as CD206, in colon tissues were enhanced by M2-EVs (Fig. 8F). Together, M2-EVs induced M2 macrophage polarization and protected tight junctions in the colons of colitis mice, in support of the in vitro functions we identified or validated in cell culture.

In a recent study [46], Wang et al. reported that M2-EVs from mouse peritoneal macrophages reduced immune cell infiltration and reserved colon length in DSS-induced colitis mice, which allied to our findings. However, the study’s authors proposed a different mechanism: M2-EVs increased the level of a long non-coding RNA (lncRNA), maternally expressed 3 (MEG3), in colon tissues, which in turn competitively bound to miR-20b-5p to promote expression of the *Creb1* gene. In our colitis mouse model, we did not observe any impact of M2-EV treatment on levels of the *Meg3* and *Creb1* gene in colon tissues (Additional file 1: Fig. S15). This mechanistic discrepancy may be due to the different experimental settings. While we gave 1.5% DSS in drinking water for 7 days and intravenously injected M2-EVs (from BMDMs, at 3 × 10^8^/g) 3 days before, on the starting date, and 2 days after the administration of DSS, Wang et al. [46] administered 5% DSS in drinking water for 7 days, followed by distilled water for 3 days and intravenously injected M2-EVs (from mouse peritoneal macrophages, at 50 μg/mouse) every 2 days.

## Discussion

In summary, our comparative proteomics analyses demonstrated the overall protein landscape of M1- and M2-EVs. The proteins uniquely enriched in M1- and M2-EVs contribute to their M1-like and M2-like phenotypes, respectively. The protein signatures of macrophage-derived EVs partially overlapped with those of their parental cells and covered many of the typical pathways and functions of their donor macrophages. Our experimental examinations validated the previous finding [9] that protein cargos in M2-EVs induced M2 polarization. More importantly, guided by the results of bioinformatic analyses, we demonstrated that signature proteins in M1-EVs were critically involved in stimulating M1 polarization and fostering viability, proliferation, and activation of T lymphocytes. In addition, we elucidated a new function of M2-EV proteins in maintaining tight junction structure and barrier integrity. In a DSS-induced colitis mouse model, intravenous injection of M2-EVs led to their accumulation in the colon, alleviation of colonic inflammation, promotion of M2 macrophage polarization, and improvement of gut barrier functions. Protein cargos in M2-EVs critically contributed to their protective functions in colitis.

Macrophage-derived EVs have been shown to exert diverse functions in immunoregulation and disease progression, which have been ascribed primarily to their microRNA cargos and occasionally to lncRNAs or individual proteins [8]. For instance, adipose tissue macrophages in obese mice were in a pro-inflammatory M1-like state, and their EVs led to insulin resistance when administered to lean mice, an effect attributed to miR-155 and miR-29a cargos [47, 48]. Tumor-associated macrophages (TAMs) are important cells that release cytokines, chemokines, and growth factors to create an immunosuppressive milieu to facilitate tumor progression and metastasis [49]. In one study of epithelial ovarian cancer [50], TAM-derived EVs were enriched with miR-21-5p and miR-29a-3p, which directly suppressed signal transducer and activator of transcription 3 (STAT3) signaling and induced an imbalance of Treg/Th17 cells to promote metastasis. In pancreatic ductal adenocarcinoma, TAM-derived EVs transferred miR-501-3p to inhibit expression of the tumor suppressor *TGFBR3* gene, thus stimulating tumor growth and invasion [51]. Incubation of M1 peritoneal macrophages with M2-EVs suppressed expression of the *Il6* and *Tnf* genes, which was partially reversed by the inhibitors of miR-23b-3p, miR-532-5p, miR-6238, and miR-let-7b-5p [52]. In another study [53], M2-EVs were found to contain a high level of lncRNA ASLNCS5088. In the recipient fibroblasts, this lncRNA bound to microRNA-200c-3p and suppressed its function, leading to increased expression of microRNA-200c-3p target genes. EVs from naïve M0 BMDMs were found to contain WNT proteins, which enhanced regenerative responses in the intestine following radiation injury [10]. A study of M2-EVs [9] showed that these vesicles, compared to M1-EVs, were enriched with a few chemokine proteins, such as C–C motif chemokine (CCL) 24 and CCL22. These chemokine protein cargos in M2-EVs contributed to their ability to convert M1 macrophages into M2 macrophages. All these studies underscore the importance of macrophage-derived EVs as couriers for intercellular communication and the functional involvement of their various cargos to regulate surrounding cells and tissues.

Although some individual protein cargos, such as WNTs and chemokines, have been shown to play critical roles in the functions of macrophage-derived EVs, comprehensive unbiased information on protein cargos in these EVs has been lacking. Our study is the first to conduct comparative bioinformatic analyses using quantitative proteome profiles of macrophage-derived EVs to yield an unprecedented rich dataset of protein cargos and a systemic view of their potential functions. Through analyses via pathway enrichment, PPI network, GSEA, and PSEA-Quant (Fig. 2, 3 and Additional file 1: Fig. S3, 4), we found that the proteins of M1-EVs were involved in proinflammatory pathways and that M2-EVs were enriched with proteins associated with immunomodulation and tissue remodeling, simulating the distinct functions of their respective parental cells. To understand the extent of the kinship of macrophages and their EVs, we conducted further comparative analyses of quantitative proteome profiles of macrophages and their EVs. This analysis, together with our immunoblot results, indicated that Cxcl10 and Arg1 could potentially serve as new specific protein markers for M1- and M2-EVs, respectively. Despite limited overlap of signature proteins of macrophage-derived EVs with their parental macrophages, pathway enrichment analysis (Fig. 4) indicated that these EVs shared a high number of pathways with their parental macrophages and carried the typical functions of their parental cells. Although it is conceivable that EVs can fulfill the typical roles of their parental cells, our comparative bioinformatic analyses provided systemic evidence of the functional resemblance between EVs and their parental cells. Our study also showcased the importance of protein cargos in EVs in carrying out the functions of their progenitor macrophages, which have been understudied and underappreciated in the macrophage EV area.

Our comparative bioinformatic analyses, combined with experimental examinations, not only validated previous findings about M2-EV protein-mediated induction of M2 polarization [9] and the engagement of M1-EVs in M1 polarization [24] and T lymphocyte activation [25], but also elucidated the importance of signature proteins in M1-EVs in M1 macrophage activation and T cell viability, proliferation, and activation (Fig. 5). More importantly, our integrative strategy revealed a completely new function of M2-EVs—they protected tight junction structure and barrier integrity from disruption through their FMOD, MFGE8, and possibly other protein cargos (Fig. 6). A previous study [54] showed that coculturing M1 macrophages with epithelial cell layers reduced TEER and disrupted the mesh structure of tight junction proteins, suggesting compromised epithelial integrity. Coculturing M2 macrophages with epithelial cells had marginal effects on TEER, but mildly disturbed the tight junction mesh structure. Thus, it seems that M2-EVs played a unique role in protecting tight junction structure and barrier integrity. Notably, our comparative analysis of M2-EVs and M2 macrophages did reveal an exclusive high enrichment of ECM organization-related proteins in M2-EVs, compared to their parental cells (Fig. 4).

Lastly, guided by our bioinformatic analyses and cell culture data, we demonstrated that M2-EVs had therapeutic potential in colitis management. Defects in intestinal macrophages in the switch from the pro-inflammatory M1-like state to the pro-resolving M2-like state are believed to significantly contribute to chronic inflammation in IBD [55, 56]. Many known IBD susceptibility loci are found in the promoter regions of LPS-regulated genes [57]. In IBD patients, intestinal macrophages often manifest a M1-like phenotype by secreting excess pro-inflammatory cytokines, promoting inflammatory responses and aggravating tissue damage [55, 56]. Therefore, macrophages, especially in their polarization states, have been regarded as novel therapeutic targets to treat colitis. M2-EVs have the inherent capability of switching M1 9 and naïve macrophages (Additional file 1: Fig. S10) to the M2 state and protecting tight junction integrity (Fig. 6). These dual functions of M2-EVs enable them to act effectively to alleviate colonic inflammation and improve gut barrier functions in the DSS-induced colitis mouse model (Fig. 7–8, Additional file 1: Fig. S13). Of note, the recent findings of an independent study [46] supported the protective functions of M2-EVs in colitis, although they proposed a different underlying mechanism. As mentioned earlier, such mechanistic differences may reflect different experimental conditions of the two groups. Nevertheless, our results together supported the view that M2-EVs represented a promising new modality to curb colitis.

## Materials and methods

### Cell culture

Bone marrow cells were isolated from femur and tibia bones of C57BL/6J mice and differentiated into BMDMs using RPMI 1640 medium (Corning, Tewksbury, MA, USA) with 10% fetal bovine serum (FBS; Atlanta Biologicals, Flowery Branch, GA, USA, S1150), 2 mM L-glutamine (Corning), 50 µg/mL penicillin/streptomycin (Corning), 1 mM sodium pyruvate (Corning), 10 mM HEPES buffer (Corning), 50 µM beta-mercaptoethanol (MilliporeSigma, St. Louis, MO, USA), and 25% L929 cell-conditioned medium or 20 ng/ml recombinant macrophage colony-stimulating factor (ProteinTech, Rosemont, IL, USA) as previously described [58]. Naïve BMDMs were incubated with 10 ng/mL of LPS (InvivoGen, San Diego, CA, USA, tlrl-peklps) for 8 h to induce M1 polarization or with 20 ng/mL of IL-4 (PeproTech, Cranbury, NJ, USA) for 24 h to induce M2 polarization. Alternatively, naïve BMDMs were incubated with 1.5 − 6 × 10^9^/mL of M1- and M2-EVs for 8 h or 24 h, respectively, to assess the polarization effects of M1- and M2-EVs.

Splenocytes were isolated from C57BL/6J mice and cultured in RPMI 1640 medium supplemented with 10% FBS, 2 mM L-glutamine, 50 µg/mL penicillin/streptomycin, and 50 µM beta-mercaptoethanol. During culture, splenocytes were treated with M1-EVs in the absence or presence of 1 µg/mL anti-CD3e antibody (ThermoFisher, Waltham, MA, USA, 16–0031-86) for 72 h to stimulate T lymphocytes.

Caco-2 cells were cultured in EMEM medium (ATCC, Manassas, VA, USA) supplemented with 10% FBS, 2 mM L-glutamine, and 50 µg/mL penicillin/streptomycin. When Caco-2 cells reached confluence, they were cultured for an additional 14 days to allow differentiation and formation of the epithelial monolayer. The culture medium was changed every three days. Afterward, the Caco-2 monolayers were treated with M2-EVs, heated M2-EVs (incubated at 95 °C for 10 min), recombinant FMOD (SinoBiological, Wayne, PA, USA), or recombinant MFGE8 (SinoBiological) for 48 h, in the presence of 1% (w/v) DSS (ThermoFisher). The TEER assay was conducted as previously described [59]. Briefly, Caco-2 cells were seeded and cultured in 24-well Transwell inserts (Corning, 6.5 mm polycarbonate membrane, 0.4 μm pore, 0.33 cm^2^ surface area). After the cells were differentiated for 14 days, they were pretreated with M2-EVs, heated M2-EVs, recombinant FMOD, or MFGE8 for 36 h, followed by treatment of 1.5% (w/v) DSS in the apical chamber for 48 h. TEER was measured using a Millicell ERS-2 voltohmmeter (MilliporeSigma). TEER value was expressed as Ω (resistance) x cm^2^ (surface area of the insert) after subtracting the blank resistance values of inserts without cells.

### Mice

C57BL/6J mice were bred and maintained in an animal facility free of specific pathogens. Animal experiments were approved by the Institutional Animal Care and Use Committee of University of Nebraska-Lincoln (Project ID 1936). To induce acute colitis, 8-week-old male C57BL/6J mice were given 1.5% (w/v) DSS in their drinking water for 7 days. Three doses of regular or heat-treated M2-EVs at 3 × 10^8^/g were intravenously injected in mice 3 days before, on the starting date, and 2 days after the administration of DSS. After 7 days of DSS treatment, the mice were sacrificed and their colon lengths were recorded. Colon segments were snap-frozen with liquid nitrogen or fixed in 10% formalin solution (VWR, Radnor, PA, USA). Pieces of colon (approximately 1 cm long) were extensively washed in PBS and ex vivo cultured in RPMI 1640 medium supplemented with 10% FBS and 50 µg/mL penicillin/streptomycin for 24 h to measure their cytokine release.

### EV preparation and characterization

EVs were isolated from the culture media of BMDMs using the standard ultracentrifugation method [12]. Before induction of macrophage polarization, BMDMs were switched to culture media with EV-free FBS. After BMDMs were treated with LPS or IL-4, the culture media were collected and subjected to sequential centrifugations at 500 ×*g* for 10 min, 2000 ×*g* for 20 min, and 10,000 ×*g* for 30 min at 4 °C. The final supernatant was subjected to ultracentrifugation at 100,000 ×*g* for 2 h at 4 °C. The EV pellet was washed, resuspended in PBS (Corning), and passed through a 200 nm syringe filter (VWR). The sizes and yields of EVs were measured using a NanoSight NS300 instrument (Malvern, Westborough, MA, USA). The protein concentration of EVs was measured using the Micro BCA Protein Assay kit (ThermoFisher). For TEM examination, a drop of macrophage-derived EVs in PBS (approximately 20 μL) was placed on parafilm and covered with a carbon-formvar coated copper grid for 30–60 s. The excess liquid was removed by touching filter paper to the edge of the grid. The samples on the grid were air dried for 30–60 s, stained in 1% uranyl acetate staining solution for 30 s, and eventually air dried. TEM images were taken at 80 kV under a Hitachi H7800 TEM using a AMT high resolution digital camera (Schaumburg, IL, USA).

### Quantitative proteomics analysis

Three biological replicates of M0-, M1-, and M2-EVs and their respective parental macrophages were independently prepared and subjected to quantitative proteomics analysis. To recover the proteins from the EV pellets, RIPA buffer was added to extract the proteins, which were reduced with 5 mM dithiothreitol, alkylated with 15 mM iodoacetamide, precipitated, and washed using the Calbiochem ProteoExtract® Protein Precipitation Kit (MilliporeSigma). Protein pellets were digested in 50 mM ammonium using trypsin (1:50). Resulting peptides were analyzed with liquid chromatography-mass spectrometry (LC–MS)/MS using a RSLCnano system (ThermoFisher) coupled to a Q-Exactive HF mass spectrometer (ThermoFisher). Separation was performed on a C18 nano column (ACQUITY UPLC M-class, Peptide CSH, 130A, 1.7 µm, 75 µm × 250 mm; Waters Corp, Milford, MA, USA) at 260 nL/min with a linear gradient from 5 to 32% B over 96 min; solvent B was 80% acetonitrile, 0.1% formic acid. For the MS1 peptide measurements, mass spectra for the eluted peptides were acquired in the data-dependent mode using a mass range of m/z 375–1500, resolution 120,000, Automatic Gain Control (AGC) target 3 × 10^6^, maximum injection time 60 ms. Data-dependent MS2 spectra were acquired by higher energy collisional dissociation as a Top10 experiment with normalized collision energy (NCE) set at 28%; AGC target was set to 1 × 10^5^, 30,000 resolution, intensity threshold 1 × 10^5^, and a maximum injection time of 200 ms. Dynamic exclusion was set at 30 s and the isolation window set to 1.6 m/z.

The proteins from macrophages were run using a Thermo Orbitrap Eclipse LC–MS/MS with online RSLCnano system running the same column configuration as for the EVs, but at 300 nL/min with a linear gradient from 3 to 22% B over 96 min; solvent B was 100% acetonitrile, 0.1% formic acid. Mass spectra for the macrophage eluted peptides were acquired as previously described for the EV peptides, except an AGC target of 4 × 10^5^ with maximum injection time of 50 ms was used for the MS1 peptide measurements; NCE was set at 30% and AGC target was set to 5 × 10^4^, 15,000 resolution with maximum injection time of 54 ms for MS2 spectra. Dynamic exclusion was set at 60 s and the isolation window set to 0.7 m/z using a top speed method with a cycle time of 3 s for data-dependent acquisition.

Acquired mass spectra were analyzed in Proteome Discoverer (ThermoFisher, v2.2 for the EV samples and v2.4 for the macrophage samples). The data were searched against the common contaminants database cRAP (116 entries, www.theGPM.org) and the Uniprot mouse database (version 20,180,720 for EV samples and version 20,221,103, 55,311 sequences for macrophage samples) using Mascot 2.6.2. Asparagine and glutamine deamidation and methionine oxidation were set as variable modifications, while Cys carbamidomethylation was specified as a fixed modification. A maximum of three trypsin missed cleavages were permitted and the precursor and fragment mass tolerances were set to 10 ppm and 0.06 Da, respectively. Peptides were validated by Percolator with a 0.01 posterior error probability (PEP) threshold. The data were searched using a decoy database to set the false discovery rate to 1% (high confidence).

The peptides were quantified using the precursor abundance based on intensity. The peak abundance was normalized using total peptide amount. Peptide group abundances were summed for each sample, and the maximum sum for all files was determined. The normalization factor was the factor of the sum of the sample and the maximum sum in all files. Protein ratios were calculated using summed abundance for each replicate separately, and the geometric median of the resulting ratios was used as the protein ratio. The significance of differential expression was tested using a t-test, which provided a *P*-value for all the calculated ratios. The normalized abundance was subjected to PCA using Clustvis [60].

### Comparative analyses of proteome profiles

ImageGP was used to generate volcano plots demonstrating proteins in M1- or M2-EVs whose levels markedly differed from those in M0-EVs [61]. Proteins whose abundances in M1- or M2-EVs were at least 1.5-fold higher than those in M0-EVs with *P* < 0.05 were selected as their signature proteins to be used for further comparative analyses. The online platform Metascape [17] was used to identify significantly enriched terms of EV protein signatures in GO cellular processes, KEGG pathways, Reactome gene sets, and WikiPathways. PPI network analysis was conducted using the STRING database [19]. The clusters of enriched proteins in M1- or M2-EVs with (a) count in network > 5; (b) strength > 0.9; and (c) false discovery rate < 0.05 in GO terms were highlighted in the network. GSEA [20] through OmicStudio was used to analyze the enrichment of gene/protein sets in M1- or M2-EV proteins. The enriched gene sets (mouse collection) with a) normalized enrichment score > 0 and b) *P* < 0.01 were selected for visualization. Protein set enrichment analysis was conducted using the PSEA-Quant algorithm [22] to identify significantly enriched protein sets of M1- or M2-EVs. The enriched protein sets with (a) number of proteins with annotation in dataset ≥ 10; and (b) false discovery rate < 0.05 were selected for visualization.

### Flow cytometry analysis

Flow cytometry analysis was conducted as previously described [58]. BMDMs were labeled with iNOS-AlexaFluor 488 (eBioscience, San Diego, CA, USA, 53-5920-80; 1:100) or Arg1-PE antibody (eBioscience, 12-3697-80; 1:40). The splenocytes were labeled with CD4-PE antibody (eBioscience, 25-0041-82; 1:200), CD8-APC antibody (eBioscience, 47-0083-82; 1:200), Zombie Violet (BioLegend, San Diego, CA, USA; 1:400), or CFSE (ThermoFisher; 1:1000).

### IF and IHF

IF staining was carried out as previously described [58]. For IHF, cryosections were prepared from colon tissues frozen in O.C.T Compound (VWR), fixed in 4% paraformaldehyde (PFA; MilliporeSigma) at 4 °C for 8 min, and blocked in 2% bovine serum albumin (BSA; VWR) at room temperature for 30 min. The blocked sections were incubated with primary antibodies at 4 °C overnight, followed by 3-time rinses in PBS and subsequent incubation with secondary antibody at room temperature for 30 min. The slides were washed in PBS 3 times, air dried, and mounted with Prolong gold antifade mountant with SYTOX deep red (ThermoFisher). Anti-occludin rabbit antibody (ProteinTech, 27,260–1-AP; 1:400), anti-claudin1 rabbit antibody (ProteinTech, 13,050-1-AP; 1:1000), and goat anti-rabbit-Alexa Fluor-488 secondary antibody (Invitrogen, A-11008; 1:200) were used for both IF and IHF. Quantitative analysis of IF and IHF images was conducted using ImageJ software.

### IHC staining

The colon paraffin sections were dewaxed by heating at 56 °C for 45 min and soaking in xylene substitute (23,412-01, Electron Microscopy Sciences, Hatfield, PA, USA; 5 min, 3 times). The dewaxed sections were rehydrated by subsequentially soaking in 100%, 95%, 90%, 80%, 70% ethanol, and PBS (3 min, 1 time in each buffer). Antigen retrieval was carried out by sub-boiling the slides in citrate unmasking solution (10 mM sodium citrate, 0.05% Tween-20, pH 6.0) for 10 min. The cooled sections were rinsed in double-distilled H_2_O (ddH_2_O; 5 min, 2 times) and incubated with 3% H_2_O_2_ for 10 min to block endogenous peroxidase activity. After rinsing in ddH_2_O (5 min, 2 times), the sections were blocked with 5% BSA in PBS for 1 h. Diluted primary antibodies were incubated with each section overnight at 4 °C. After rinsing in Tris-buffered saline with Tween 20 (TBST), the sections were incubated with horseradish peroxidase-conjugated anti-rabbit secondary antibody SignalStain Boost IHC detection reagent (Cell Signaling, Danvers, MA, USA) for 30 min at room temperature and rinsed in TBST (5 min, 3 times). The staining color was developed using SignalStain DAB substrate kit (Cell signaling), and nucleus counterstaining was performed using Hemotoxylin solution (MilliporeSigma). After washing in TBST, the sections were dehydrated by immersing in 95% ethanol (10 s, 2 times), 100% ethanol (10 s, 2 times), and xylene (10 s, 2 times) and mounted using DPX mountant (Fisher, Hampton, NH, USA). Primary antibodies used included antibodies against iNOS (ABclonal, A0312; 1:100), CD206/Mrc1 (Cell Signaling, 24,595; 1:400), and F4/80 (Cell Signaling, 70,076; 1:200). Quantitative analysis of IHC images was carried out using ImageJ software.

### Labeling and uptake of macrophage-derived EVs in cell culture and mice

Macrophage-derived EVs were labeled with PKH26 labeling kit (MilliporeSigma) according to the manufacturer’s protocol. The fluorescence-labeled M1-EVs (4.5 × 10^9^/mL) or M2-EVs (1.5 × 10^9^/mL) were incubated with BMDMs for 8 h or 24 h, respectively. The cells were washed 3 times with PBS and fixed with 4% paraformaldehyde. Images were acquired by a A1R-Ti2 confocal microscope (Nikon, Melville, NY, USA).

The biodistribution of M2-EVs in mice was measured as described [58] with minor modifications. M2-EVs were labeled with ExoGlow-Vivo EV labeling kit (System Biosciences, Palo Alto, CA, USA) at the ratio of 2 × 10^10^ EVs to 1 μL dye following the manufacturer’s protocol. To remove the free dye, M2-EVs were washed with PBS and ultra-centrifuged at 100,000 ×*g* for 2 h at 4 °C. The labeled M2-EVs (2,500 fluorescence intensity/g) were intravenously administered to 8-week-old male C57BL/6J mice with colitis, which was induced by feeding the mice with 1.5% (w/v) DSS in their drinking water for 7 days. 6 h later, the mice were sacrificed to collect different parts of the gastrointestinal (GI) tract and a variety of tissues. For the GI tract, the contents in the lumen were removed, followed by extensive washing using PBS to remove food residue, feces, and any un-absorbed M2-EVs. The fluorescence signals of collected organs were measured using an Odyssey Clx imaging system (LI-COR Biosciences, Lincoln, NE, USA) and normalized to tissue weights.

### In vitro collagen digestion

Collagen powder (MilliporeSigma) was dissolved in PBS at 1 mg/mL and sonicated on ice for 5 min using a digital sonifier S-450D (Branson Ultrasonic, Danbury, CT, USA). The solution was centrifuged at 500 ×*g* for 10 min at 4 °C, and the supernatant was collected. M2-EVs were sonicated on ice for 5 min using the digital sonifier S-450D or lysed using lysis buffer (150 mM NaCl, 0.5% NP-40, 50 mM Tris–HCl, pH 7.5) to release protein cargos. The protein concentrations of collagen solution and M2-EV samples were quantified using the Piece BCA protein assay kit (ThermoFisher). The collagen solution was incubated with type VIII collagenase (MilliporeSigma) at 37 °C for 10 min or with sonicated/lysed M2-EVs at 37 °C overnight. The solution was mixed with SDS-gel loading buffer and heated at 95 °C for 10 min. The samples were separated on a Bis–Tris protein gel and stained with Coomassie blue.

### Enzyme-linked immunosorbent assay (ELISA), immunoblot, and mRNA analysis

ELISA, immunoblot, and mRNA analysis were conducted as described [62, 63]. ELISA kits included IFN-γ (Invitrogen, 88–8314-22), IL-1β (eBioscience, 88,701,388), IL-18 (MBL, D042-3), IL-6 (BioLegend, 431,301), and TNF-α (BioLegend, 430,901). Primary antibodies used in this study include antibodies against ALIX (Cell Signaling, 2171, 1:1000), TSG101 (Genetex, GTX70255; 1:500), GAPDH (Cell Signaling, 2118; 1:1000), CD206/MRC1 (Cell Signaling, 24,595; 1:1000), occludin (ProteinTech, 27,260-1-AP; 1:1000), claudin1 (ProteinTech, 13,050-1-AP; 1:1000), CD81 (ProteinTech, 27,855-1-AP; 1:1000), Cxcl10 (ProteinTech, 10,937-1-AP; 1:1000), Arg1 (ProteinTech, 66,129-1; 1:1000), FMOD (ProteinTech, 60,108-1; 1:500), and MFGE8 (ThermoFisher, PA5-109,955; 1:1000).

### Statistics

The data were analyzed using Excel software and a two-tailed t-test was performed. *P* < 0.05 was indicated by * and considered significant and *P* < 0.01 was indicated by **. Each cell culture experiment was repeated at least three times.

### Supplementary Information

**Additional file 1: Figure S1.** Validation of M1 and M2 BMDMs and EV characterization. **A** M1 and M2 BMDMs were validated by flow cytometry analysis using antibodies against M1 marker iNOS and M2 marker Arg1. Naïve M0 BMDMs were treated with LPS for 8 h to induce M1 polarization or treated with IL4 for 24 h to induce M2 activation. **B** Quantification of the EV yields. Data presented as mean ± SEM. N = 7–9 independent EV preparations. *P < 0.05. **C** EV purity. The purity of EVs was determined by comparing the ratio of EV yield to protein concentration. Data presented as mean ± SEM. N = 3. **Figure S2.** Comparative analyses of protein profiles of macrophage-derived EVs. **A** 79 proteins were dramatically upregulated and 38 downregulated in M1-EVs, compared to M0-EVs. **B** PCA of the protein profiles of M0- and M1-EV pairs showed a good separation of M0- and M1-EV proteins. **C** 53 proteins were significantly increased and 64 decreased in M2-EVs, compared to M0-EVs. **D** PCA showed that M2-EVs proteins were clustered together and separated from M0-EV proteins. **E** Volcano plot demonstrating the significantly upregulated and downregulated proteins in M1-EVs, based on their fold changes and *P*-values. **F** Volcano plot of M2-EV protein cargos. **Figure S3.** GSEA of total proteins in M1- and M2-EVs. The genes that encode proteins identified in M1- (**A**) or M2-EVs (**B**) were compared with the M5 ontology gene sets (mouse collection) from MSigDB in GSEA. The enriched gene sets with (a) size ≥ 15 and (b) false discovery rate < 0.1 were selected for visualization. **Figure S4.** PSEA-Quant analysis of total proteins in M1- and M2-EVs. Proteins identified in M1- (**A**) or M2-EVs (**B**) were compared with the protein sets from PSEA-Quant. The top 20 enriched protein sets with a) number of proteins with annotation in dataset ≥ 10 and b) false discovery rate < 0.05 were selected for visualization. **Figure S5.** Comparative analysis of protein signatures of M1-EVs with M1 BMDMs. The enriched terms of both M1-EVs and M1 BMDMs were combined to run the comparative analysis. Heatmap of top 100 terms demonstrating the similarities and differences of protein signatures of M1-EVs and M1 BMDMs. **Figure S6.** Comparative analysis of protein signatures of M2-EVs with M2 BMDMs. The enriched terms of both M2-EVs and M2 BMDMs were combined to run the comparative analysis. Heatmap of top 70 pathways showing the similarities and differences of protein signatures of M2-EVs and M2 BMDMs. **Figure S7.** Comparative analyses of protein signatures of M1- and M2-EVs with gene signatures of their respective parental macrophages. **A** Venn diagram showing the overlap of protein signature of M1-EVs with gene signature of M1 BMDMs. **B** Circos plot depicting the signature proteins and pathways shared between M1-EVs and M1 BMDMs. **C** Overlap of enriched proteins in M2-EVs with gene signatures of M2 BMDMs. **D** Circos plot demonstrating the mutual signature proteins and pathways of M2-EVs and M2 BMDMs. **E** Heatmap showing that M1-EV signature proteins shared 15 of the top 20 pathways of M1 BMDMs. **F** Heatmap indicating that M2-EV signature proteins shared nine of the top 18 pathways of M2 BMDMs. **G** GSEA demonstrated that M1-EVs were significantly enriched with proteins related to M1 gene signature. **H** GSEA showed that M2-EV proteins were highly correlated with M2 gene signature. **Figure S8.** M1-EVs had no ability to induce the *Arg1* gene and their uptake by macrophages. **A** Expression of the M2 marker *Arg1* gene in BMDMs treated with 3 × 10^9^/mL of different macrophage-derived EVs. **B** Heat treatment of M1-EVs did not affect their uptake by macrophages. 4.5 × 10^9^/mL of regular or heated M1-EVs were labeled with lipophilic dye PKH26 and incubated with BMDMs for 8 h. The cells were extensively washed and fixed and their images were taken using a confocal microscope. DAPI (4′,6-diamidino-2-phenylindole) was included to stain nuclei. Data was presented as mean ± STD (N = 3). ** *P* < 0.01. **Figure S9.** M1-EVs promoted cytokine release from splenocytes. Splenocytes were cultured with increasing amount of M1-EVs for 72 h in the absence of anti-CD3 antibody. Culture media were collected to measure the release of INF-γ (**A**) and TNF-α (**B**). Data was presented as mean ± STD (N = 3). **P < 0.01. **Figure S10.** Protein cargos of M2-EVs induced M2 polarization in naïve BMDMs. **A** Expression of M2 marker genes in naïve BMDMs treated with 1.5 × 10^9^/mL of M0- or M2-EVs for 24 h. **B** Expression of the M1 marker *Nos2* gene in BMDMs treated with 3 × 10^9^/mL of different macrophage-derived EVs. **C** Flow cytometry analysis demonstrated the protein levels of iNOS and Arg1 in BMDMs treated with 6 × 10^9^/mL of M0- or M2-EVs for 24 h. **D** Heat treatment of M2-EVs did not influence their uptake by macrophages. M2-EVs were heated at 95 °C for 10 min to denature their protein cargos. 1.5 × 10^9^/mL of regular or heated M2-EVs were labeled with lipophilic dye PKH26 and incubated with BMDMs for 24 h. The cells were extensively washed and fixed and their images were taken using a confocal microscope. DAPI was included to stain nuclei. **E** Protein levels of iNOS and Arg1 in BMDMs treated with 6 × 10^9^/mL of heated M0- or M2-EVs for 24 h. **F** Expression of M2 marker genes in naïve BMDMs treated with 1.5 × 10^9^/mL of regular or heated M2-EVs for 24 h. Data was presented as mean ± STD (N = 3). **P < 0.01. **Figure S11.** Proteins in M2-EVs were not able to degrade collagens. **A** 0.2 μg/μL collagen mixture solution was incubated with 0.6 μg/μL type VIII collagenase for 10 min or with sonicated M2-EVs with a protein concentration of 0.2 μg/μL for overnight at 37 °C. **B** 0.2 μg/μL collagen mixture solutions was incubated with 1 μg/μL type VIII collagenase for 10 min or with lysed M2-EVs with a protein concentration of 0.5 μg/μL overnight at 37 °C. After digestion, the protein mixture was separated on a Bis–Tris protein gel, followed by Coomassie blue staining. **Figure S12.** Claudin1 IF of Caco-2 monolayers confirmed that proteins in M2-EVs critically contributed to protection of tight junction structure. **A** Representative images of claudin1 IF of Caco-2 cells and quantification of claudin1 signal intensity. The differentiated Caco-2 monolayers were treated with PBS or regular or heated M2-EVs in PBS (6 × 10^9^/mL) for 48 h in the presence of 1% DSS. **B** Immunoblot analysis validated the enrichment of FMOD and MFGE8 proteins in M2-EVs. In each lane, 5 μg proteins from EV lysates were loaded. ALIX served as a loading control. **C** Representative images of claudin1 IF of Caco-2 cells and quantification of claudin1 signal intensity. The differentiated Caco-2 cells were treated with PBS, M2-EVs in PBS (6 × 10^9^/mL), FMOD (3 µg/mL), MFGE8 (3 µg/mL), or FMOD (1.5 µg/mL) and MFGE8 (1.5 µg/mL) together for 48 h in the presence of 1% DSS. Data was presented as mean ± STD (N = 3). *P < 0.05 and **P < 0.01. **Figure S13.** M2-EVs protected mice from DSS-induced colitis. The same experimental procedure outlined in Fig. 7A was used. 2-month-old male C57BL/6J mice were intravenously injected with PBS or M2-EVs in PBS on day 1, 4, and 6. The mice were given 1.5% (w/v) DSS in drinking water from day 4–11 and sacrificed on day 11. **A** M2-EVs prevented the shortened colon length in mice induced by DSS treatment. N = 14–15/group. **B** The levels of cytokines in the media of ex vivo cultured colonic tissues. N = 9–10/group. **C** Expression of pro-inflammatory cytokine genes in colon tissues. N = 12/group. In the bar graphs, each dot represents one mouse. Data were presented as mean ± SEM. *P < 0.05 and **P < 0.01 relative to the control colitis mice received PBS (bar with black dots). **Figure S14.** Biodistribution of intravenously injected M2-EVs in colitis mice. M2-EVs were covalently labeled with a fluorescence dye in near infrared ranges. The solution PBS or the labeled M2-EVs in PBS at 2,500 fluorescence intensity/g were intravenously administered to 2-month-old male C57BL/6J mice, which were fed with 1.5% (w/v) DSS-containing drinking water for 7 days to induce colitis. 6 h later, the mice were sacrificed and their tissues were collected to measure their fluorescence signals. N = 3/group. **A** Representative images of mouse tissues under the Licor Odyssey Clx image system. **B** Relative fluorescence signal intensity of mouse tissues. The fluorescence signal intensity of each tissue was normalized to the tissue weight. Upper gastrointestinal (GI) tract included stomach and small intestine. eWAT: epididymal white adipose tissue. BAT: Brown adipose tissue. In the bar graphs, each dot represents one mouse. Data were presented as mean ± SEM. *P < 0.05 and **P < 0.01 relative to the control colitis mice received PBS (bar with black dots). **Figure S15.** Expression level of the *Meg3* and *Creb1* genes. The mice from Fig. S13 were used to conduct qPCR to determine the expression level of the *Meg3* and *Creb1* genes. N = 6–8/group. In the bar graphs, each dot represents one mouse. Data were presented as mean ± SEM.

**Additional file 2: Table S1.** Protein profiles of M0- and M1-EV pairs.

**Additional file 3: Table S2.** Protein profiles of M0- and M2-EV pairs.

**Additional file 4: Table S3.** M1-EV protein signatures. M2-EV protein signatures.

## Data Availability

All data are included in this published article and its supplementary information files.
