# Supplementary material for: Critical contributions of protein cargos to the functions of macrophage-derived extracellular vesicles
Source: J Nanobiotechnology. 2023 Sep 28;21:352. doi: 10.1186/s12951-023-02105-9 (PMC10537535; doi:10.1186/s12951-023-02105-9)
Supplement: Supplementary file 1 — Additional file 1: Figure S1. Validation of M1 and M2 BMDMs and EV characterization. A M1 and M2 BMDMs were validated by flow cytometry analysis using antibodies against M1 marker iNOS and M2 marker Arg1. Naïve M0 BMDMs were treated with LPS for 8 h to induce M1 polarization or treated with IL4 for 24 h to induce M2 activation. B Quantification of the EV yields. Data presented as mean ± SEM. N = 7–9 independent EV preparations. *P < 0.05. C EV purity. The purity of EVs was determined by comparing the ratio of EV yield to protein concentration. Data presented as mean ± SEM. N = 3. Figure S2. Comparative analyses of protein profiles of macrophage-derived EVs. A 79 proteins were dramatically upregulated and 38 downregulated in M1-EVs, compared to M0-EVs. B PCA of the protein profiles of M0- and M1-EV pairs showed a good separation of M0- and M1-EV proteins. C 53 proteins were significantly increased and 64 decreased in M2-EVs, compared to M0-EVs. D PCA showed that M2-EVs proteins were clustered together and separated from M0-EV proteins. E Volcano plot demonstrating the significantly upregulated and downregulated proteins in M1-EVs, based on their fold changes and P-values. F Volcano plot of M2-EV protein cargos. Figure S3. GSEA of total proteins in M1- and M2-EVs. The genes that encode proteins identified in M1- (A) or M2-EVs (B) were compared with the M5 ontology gene sets (mouse collection) from MSigDB in GSEA. The enriched gene sets with (a) size ≥ 15 and (b) false discovery rate < 0.1 were selected for visualization. Figure S4. PSEA-Quant analysis of total proteins in M1- and M2-EVs. Proteins identified in M1- (A) or M2-EVs (B) were compared with the protein sets from PSEA-Quant. The top 20 enriched protein sets with a) number of proteins with annotation in dataset ≥ 10 and b) false discovery rate < 0.05 were selected for visualization. Figure S5. Comparative analysis of protein signatures of M1-EVs with M1 BMDMs. The enriched terms of both M1-EVs and M1 BMDMs wer [file 12951_2023_2105_MOESM1_ESM.docx]

| 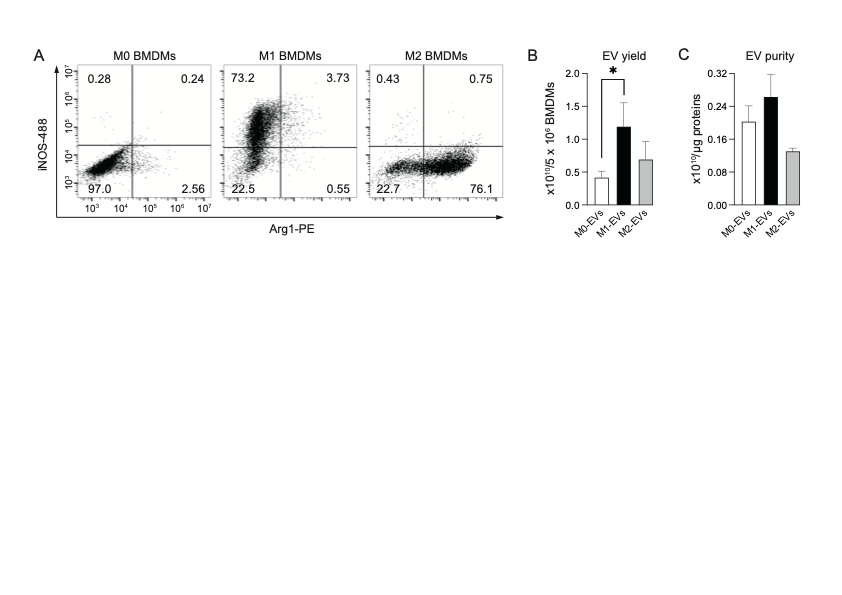 |
| --- |
| **Figure S1. Validation of M1 and M2 BMDMs and EV characterization.** (A) M1 and M2 BMDMs were validated by flow cytometry analysis using antibodies against M1 marker iNOS and M2 marker Arg1. Naïve M0 BMDMs were treated with LPS for 8 h to induce M1 polarization or treated with IL4 for 24 h to induce M2 activation. (B) Quantification of the EV yields. Data presented as mean±SEM. N=7-9 independent EV preparations. * *P* < 0.05. (C) EV purity. The purity of EVs was determined by comparing the ratio of EV yield to protein concentration. Data presented as mean±SEM. N=3. |

|  |
| --- |

**Figure S2.** **Comparative analyses of protein profiles of macrophage-derived EVs.** (A) 79 proteins were dramatically upregulated and 38 downregulated in M1-EVs, compared to M0-EVs. (B) PCA of the protein profiles of M0- and M1-EV pairs showed a good separation of M0- and M1-EV proteins. (C) 53 proteins were significantly increased and 64 decreased in M2-EVs, compared to M0-EVs. (D) PCA showed that M2-EVs proteins were clustered together and separated from M0-EV proteins. (E) Volcano plot demonstrating the significantly upregulated and downregulated proteins in M1-EVs, based on their fold changes and *P*-values. (F) Volcano plot of M2-EV protein cargos.

|  |
| --- |
| **Figure S3. GSEA of total proteins in M1- and M2-EVs.**The genes that encode proteins identified in M1- (A) or M2-EVs (B) were compared with the M5 ontology gene sets (mouse collection) from MSigDB in GSEA. The enriched gene sets with a) size ≥ 15 and b) false discovery rate < 0.1 were selected for visualization. |

| ****  **Figure S4. PSEA-Quant analysis of total proteins in M1- and M2-EVs.**Proteins identified in M1- (A) or M2-EVs (B) were compared with the protein sets from PSEA-Quant. The top 20 enriched protein sets with a) number of proteins with annotation in dataset ≥ 10 and b) false discovery rate < 0.05 were selected for visualization. |
| --- |

|  |
| --- |
| **Figure S5. Comparative analysis of protein signatures of M1-EVs with M1 BMDMs.** The enriched terms of both M1-EVs and M1 BMDMs were combined to run the comparative analysis. Heatmap of top 100 terms demonstrating the similarities and differences of protein signatures of M1-EVs and M1 BMDMs. |

| ****  **Figure S6. Comparative analysis of protein signatures of M2-EVs with M2 BMDMs.** The enriched terms of both M2-EVs and M2 BMDMs were combined to run the comparative analysis. Heatmap of top 70 pathways showing the similarities and differences of protein signatures of M2-EVs and M2 BMDMs. |
| --- |
|  |
| ****  **Figure S7. Comparative analyses of protein signatures of M1- and M2-EVs with gene signatures of their respective parental macrophages**. (A) Venn diagram showing the overlap of protein signature of M1-EVs with gene signature of M1 BMDMs. (B) Circos plot depicting the signature proteins and pathways shared between M1-EVs and M1 BMDMs. (C) Overlap of enriched proteins in M2-EVs with gene signatures of M2 BMDMs. (D) Circos plot demonstrating the mutual signature proteins and pathways of M2-EVs and M2 BMDMs. (E) Heatmap showing that M1-EV signature proteins shared 15 of the top 20 pathways of M1 BMDMs. (F) Heatmap indicating that M2-EV signature proteins shared nine of the top 18 pathways of M2 BMDMs. (G) GSEA demonstrated that M1-EVs were significantly enriched with proteins related to M1 gene signature. (H) GSEA showed that M2-EV proteins were highly correlated with M2 gene signature. |

|  |
| --- |
| **Figure S8. M1-EVs had no ability to induce the *Arg1* gene and their uptake by macrophages.** (A) Expression of the M2 marker *Arg1* gene in BMDMs treated with 3×10^9^/mL of different macrophage-derived EVs. (B) Heat treatment of M1-EVs did not affect their uptake by macrophages. 4.5×10^9^/mL of regular or heated M1-EVs were labeled with lipophilic dye PKH26 and incubated with BMDMs for 8 h. The cells were extensively washed and fixed and their images were taken using a confocal microscope. DAPI (4′,6-diamidino-2-phenylindole) was included to stain nuclei. Data was presented as mean±STD (N=3). ** *P* <0.01. |

|  |
| --- |
| **Figure S9. M1-EVs promoted cytokine release from splenocytes.** Splenocytes were cultured with increasing amount of M1-EVs for 72 h in the absence of anti-CD3 antibody. Culture media were collected to measure the release of INF-γ (A) and TNF-α (B). Data was presented as mean±STD (N=3). ** *P* <0.01. |

|  |
| --- |
| **Figure S10. Protein cargos of M2-EVs induced M2 polarization in naïve BMDMs.** (A) Expression of M2 marker genes in naïve BMDMs treated with 1.5×10^9^/mL of M0- or M2-EVs for 24 h. (B) Expression of the M1 marker *Nos2* gene in BMDMs treated with 3×10^9^/mL of different macrophage-derived EVs. (C) Flow cytometry analysis demonstrated the protein levels of iNOS and Arg1 in BMDMs treated with 6×10^9^/mL of M0- or M2-EVs for 24 h. (D) Heat treatment of M2-EVs did not influence their uptake by macrophages. M2-EVs were heated at 95 °C for 10 min to denature their protein cargos. 1.5×10^9^/mL of regular or heated M2-EVs were labeled with lipophilic dye PKH26 and incubated with BMDMs for 24 h. The cells were extensively washed and fixed and their images were taken using a confocal microscope. DAPI was included to stain nuclei. (E) Protein levels of iNOS and Arg1 in BMDMs treated with 6×10^9^/mL of heated M0- or M2-EVs for 24 h. (F) Expression of M2 marker genes in naïve BMDMs treated with 1.5×10^9^/mL of regular or heated M2-EVs for 24 h. Data was presented as mean±STD (N=3). ** *P* <0.01. |

|  |
| --- |
| **Figure S11. Proteins in M2-EVs were not able to degrade collagens and validation of two protein cargos in M2-EVs.** (A and B) Proteins in M2-EVs were not able to degrade collagens. (A) 0.2 μg/μL collagen mixture solution was incubated with 0.6 μg/μL type VIII collagenase for 10 min or with sonicated M2-EVs with a protein concentration of 0.2 μg/μL for overnight at 37 °C. (B) 0.2 μg/μL collagen mixture solutions was incubated with 1 μg/μL type VIII collagenase for 10 min or with lysed M2-EVs with a protein concentration of 0.5 μg/μL overnight at 37 °C. After digestion, the protein mixture was separated on a Bis-Tris protein gel, followed by Coomassie blue staining. |

|  |
| --- |
| **Figure S12. Claudin1 IF of Caco-2 monolayers confirmed that proteins in M2-EVs critically contributed to protection of tight junction structure.** (A) Representative images of claudin1 IF of Caco-2 cells and quantification of claudin1 signal intensity. The differentiated Caco-2 monolayers were treated with PBS or regular or heated M2-EVs in PBS (6×10^9^/mL) for 48 h in the presence of 1% DSS. (B) Immunoblot analysis validated the enrichment of FMOD and MFGE8 proteins in M2-EVs. In each lane, 5 μg proteins from EV lysates were loaded. ALIX served as a loading control. (C) Representative images of claudin1 IF of Caco-2 cells and quantification of claudin1 signal intensity. The differentiated Caco-2 cells were treated with PBS, M2-EVs in PBS (6×10^9^/mL), FMOD (3 µg/mL), MFGE8 (3 µg/mL), or FMOD (1.5 µg/mL) and MFGE8 (1.5 µg/mL) together for 48 h in the presence of 1% DSS. Data was presented as mean±STD (N=3). * *P* <0.05 and ** *P* <0.01. |

|  |
| --- |
| **Figure S13. M2-EVs protected mice from DSS-induced colitis.** The same experimental procedure outlined in Figure 7A was used. 2-month-old male C57BL/6J mice were intravenously injected with PBS or M2-EVs in PBS on day 1, 4, and 6. The mice were given 1.5% (w/v) DSS in drinking water from day 4-11 and sacrificed on day 11. (A) M2-EVs prevented the shortened colon length in mice induced by DSS treatment. N=14-15/group. (B) The levels of cytokines in the media of *ex vivo* cultured colonic tissues. N=9-10/group. (C) Expression of pro-inflammatory cytokine genes in colon tissues. N=12/group. In the bar graphs, each dot represents one mouse. Data were presented as mean±SEM. * *P* < 0.05 and ** *P* < 0.01 relative to the control colitis mice received PBS (bar with black dots). |

|  |
| --- |
| **Figure S14. Biodistribution of intravenously injected M2-EVs in colitis mice.** M2-EVs were covalently labeled with a fluorescence dye in near infrared ranges. The solution PBS or the labeled M2-EVs in PBS at 2,500 fluorescence intensity/g were intravenously administered to 2-month-old male C57BL/6J mice, which were fed with 1.5% (w/v) DSS-containing drinking water for 7 days to induce colitis. 6 h later, the mice were sacrificed and their tissues were collected to measure their fluorescence signals. N = 3/group. (A) Representative images of mouse tissues under the Licor Odyssey Clx image system. (B) Relative fluorescence signal intensity of mouse tissues. The fluorescence signal intensity of each tissue was normalized to the tissue weight. Upper gastrointestinal (GI) tract included stomach and small intestine. eWAT: epididymal white adipose tissue. BAT: Brown adipose tissue. In the bar graphs, each dot represents one mouse. Data were presented as mean±SEM. * *P* < 0.05 and ** *P* < 0.01 relative to the control colitis mice received PBS (bar with black dots). |

|  |
| --- |
| **Figure S15. Expression level of the *Meg3* and *Creb1* genes.** The mice from Figure S13 were used to conduct qPCR to determine the expression level of the *Meg3* and *Creb1* genes. N= 6-8/group. In the bar graphs, each dot represents one mouse. Data were presented as mean±SEM. |
